# Very Low Population Structure in a Highly Mobile and Wide-Ranging Endangered Bird Species

**DOI:** 10.1371/journal.pone.0143746

**Published:** 2015-12-09

**Authors:** Lynna Kvistad, Dean Ingwersen, Alexandra Pavlova, James K. Bull, Paul Sunnucks

**Affiliations:** 1 School of Biological Sciences, Monash University, Melbourne, Victoria, Australia; 2 BirdLife Australia, Melbourne, Victoria, Australia; University of British Columbia Okanagan, CANADA

## Abstract

The loss of biodiversity following fragmentation and degradation of habitat is a major issue in conservation biology. As competition for resources increases following habitat loss and fragmentation, severe population declines may occur even in common, highly mobile species; such demographic decline may cause changes within the population structure of the species. The regent honeyeater, *Anthochaera phrygia*, is a highly nomadic woodland bird once common in its native southeast Australia. It has experienced a sharp decline in abundance since the late 1970s, following clearing of large areas of its preferred habitat, box-ironbark woodland, within the last 200 years. A captive breeding program has been established as part of efforts to restore this species. This study used genetic data to examine the range-wide population structure of regent honeyeaters, including spatial structure, its change through time, sex differences in philopatry and mobility, and genetic differences between the captive and wild populations. There was low genetic differentiation between birds captured in different geographic areas. Despite the recent demographic decline, low spatial structure appears to have some temporal consistency. Both sexes appear to be highly mobile, and there does not seem to be significant genetic differentiation between the captive and wild populations. We conclude that management efforts for survival of this species, including habitat protection, restoration, and release of captive-bred birds into the wild, can treat the species as effectively a single genetic population.

## Introduction

Habitat clearance is a major global issue and a key factor in biodiversity decline [1]. A decrease in the overall amount of habitat leads to an increase in competition for scarcer resources, while fragmentation of remaining suitable habitat disrupts ecological processes including movement and dispersal of organisms [2]. Reduced connectivity may disrupt metapopulation dynamics, causing formerly connected populations to become isolated; this may lead to genetic drift and loss of genetic diversity, with limited opportunity for gene flow [3]. Limited connectivity may also reduce the probability of patch persistence as small subpopulations without an influx of immigrants are at higher risk of demographic and genetic stochasticity than larger populations, thus placing populations of species affected by fragmentation at risk of extinction [4–7]. Demographic decline may then lag behind the initial habitat loss as deterministic effects unfold so that eventual extinction can occur years later [8]. While highly mobile species may have strong ability to disperse between patches and so are predicted to have greater genetic connectivity in fragmented habitats than are less mobile species, they may also require a larger range of resources, which can be adversely affected by habitat loss [9–10]. Dependence upon landscape-level resources may negatively affect abundance if such resources fail [11].

To understand the effects of loss of habitat and associated fragmentation on biodiversity, such as how fragmentation creates or affects population structure, the time it takes for population structure to change following habitat alteration, and if fragmentation differentially affects sexes, it may be particularly useful to use woodlands as study systems. Woodlands have been preferentially cleared throughout the world due to their high value to humans as sources of timber and fertile soil for agriculture [12, 1]. Australian woodland birds present an unusual and powerful model for studying the effects of habitat loss on biodiversity because such large-scale woodland clearing has occurred only within the last two centuries following the arrival of European-style industrialization; this clearance has been strong and rapid, resulting in the severe reduction and fragmentation of woodland habitat [13, 11, 14]. The species diversity within the Australian avifauna offers the potential to study the impacts of habitat loss on species with a variety of traits that may affect their ability to survive in a highly fragmented environment, ranging from species that move large distances to find resources on a continent with volatile ecology to much more static species that have traded dispersal ability for competition. While the processes causing declines of sedentary birds in fragmented landscapes may be explained by reduced dispersal and gene flow between patches, the mechanisms underlying declines of mobile species are less understood [9, 15].

The regent honeyeater, *Anthochaera phrygia*, is a highly mobile, nectarivorous bird that has undergone a severe demographic decline following the extensive clearing of woodlands in southeast Australia [16]. Here, the most fertile land was cleared for agriculture, leaving behind mostly lower quality remnant vegetation [17, 18]. Once an abundant and widespread species, regent honeyeaters are detailed in nineteenth century accounts as frequently occurring in flocks of thousands along the country’s southeast coast between southeastern Queensland and southeastern South Australia [16]. However, population decline was observed in the late 1970s; current estimates suggest only around 400 adult regent honeyeaters remain in the wild, and the species is largely restricted to New South Wales [16, 19, 20, 18]. The delay between major clearing and demographic decline may be due to fulfillment of an extinction debt caused by resource scarcity and increased competition with other birds [10, 18].

To help increase the regent honeyeater’s chance of survival, a captive breeding program for the species has been in operation at Taronga Zoo in Sydney since 1995 [21]. In an effort to increase the number of wild birds, 109 captive-bred individuals were released into the wild between 2008 and 2013 in Chiltern–Mt Pilot National Park in Victoria [22]. Although monitoring of reproduction of the species in the field is challenging and events such as breeding are necessarily rare due to their low occurrence, breeding attempts between wild and captive birds have been observed, and a released pair has successfully raised two chicks to fledging stage [22]. Understanding if there is population structure in regent honeyeaters will aid the recovery effort by informing if the species may be managed as one unit.

The existence of population structure in regent honeyeaters would indicate that the population operates as multiple subpopulations rather than a larger, more homogeneous unit; the presence of small, genetically isolated subpopulations would suggest that this threatened species is facing increased risks of demographic and genetic stochastic effects. Although the regent honeyeater has evolved to be highly mobile so that it is able to constantly access rich nectar sources, such mobility does not immediately equate to the occurrence of gene flow between patches: other wide-ranging species with high dispersal ability can nevertheless have genetically distinct localized subunits [16, 23–25].

While habitat fragmentation often has impacts on the population genetic structure of bird species, the extent, direction, and onset can be unpredictable. While fragmentation may, in some circumstances, prevent birds from dispersing between patches, in others it may require them to disperse farther than what was necessary in more continuous habitat [9, 26]. Responses to habitat fragmentation can also have strong temporal lags in some species. Because regent honeyeaters can survive at least 10 years in the wild, it is possible that the decline of this species has only been for a few generations and it has thus far been able to avoid extensive loss of genetic diversity [27, 28]. It is also becoming increasingly clear that sex differences in wildlife responses to habitat fragmentation can be an important factor in demographic impacts. If one sex has greater dispersal ability than the other, movement and gene flow between patches can be mostly dependent on the more mobile sex. This may translate into demographic decline if patch emigration exceeds immigration, causing members of the more sedentary sex to become unable to reliably find mates and reproduce, and can generate sex-biased genetic differences [9, 29–31]. By analyzing genotypic data (10 polymorphic microsatellite loci) for regent honeyeaters, this study tests for i) presence of population substructure; ii) temporal changes in genetic diversity; iii) differences in mobility between sexes; and iv) genetic differentiation between the captive and wild populations.

## Methods

### Study species

The regent honeyeater is a medium-sized (38–50 g) honeyeater native to southeastern Australia [32]. The species is largely nectarivorous, although arthropods, lerp (a carbohydrate produced by psyllid insects), and manna (a carbohydrate produced by plants) are also important dietary components [18, 32, 33–35]. Box-ironbark woodland is the preferred habitat of regent honeyeaters, although this species also occurs in box-gum, box-stringybark, dry plateau woodland, coastal swamp mahogany forest, and riparian gallery forest [36–39].

### Study region and sampling

**Fig 1:**
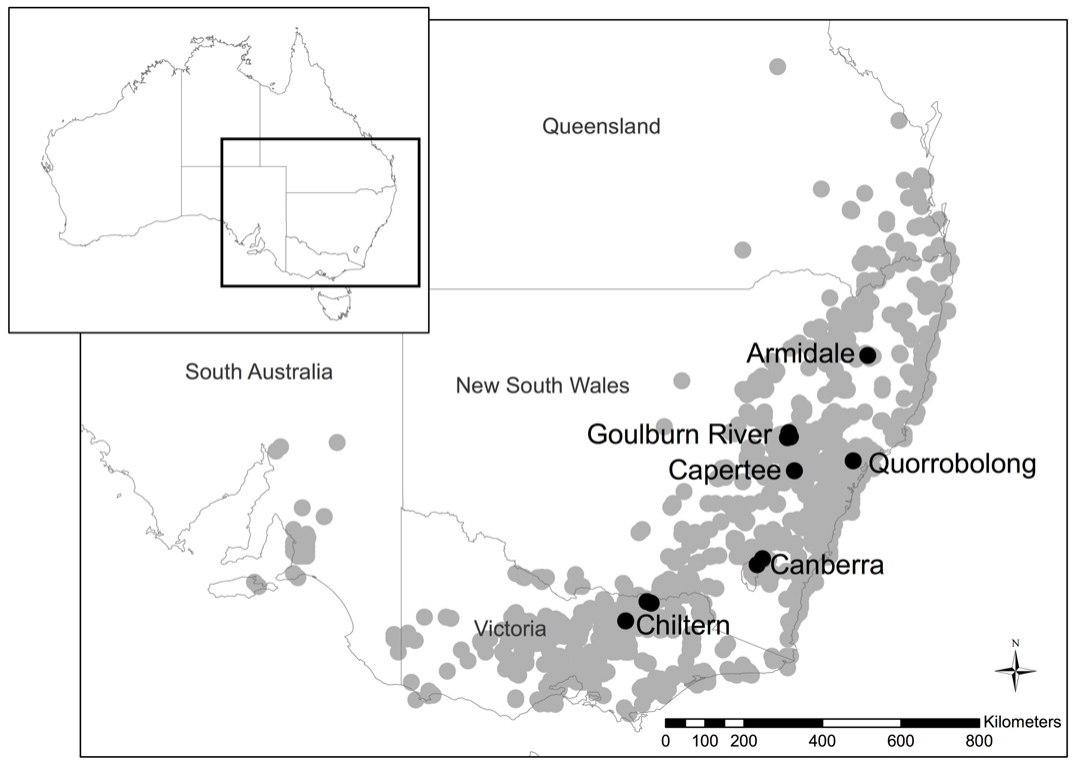
Map depicting sampling sites (black dots) within the historical range of regent honeyeaters. Gray dots indicate 2227 geographic sites for 4542 observational records of regent honeyeaters, data taken from Atlas of Living Australia [40].

Birds were captured using call playback and mist nets. Blood (up to 50 μl) and feather samples from wild adults and juveniles were taken at several locations in southeast Australia (Table 1, Fig 1) in all seasons, between 1989 and 2012 (S1 Table). Blood samples were obtained from the brachial vein; after puncturing the vein with a narrow gauge needle, blood was collected into microcapillary tubes and aspirated into 96%+ ethanol, then refrigerated until transferred to a -20°C freezer. Blood and feather samples from captive birds were taken between 2010 and 2013. Blood from the jugular vein was collected while birds were under anesthesia via isoflurane; samples were then placed in 96%+ ethanol until transferred to a -20° C freezer. The regent honeyeater is critically endangered under the Environment Protection and Conservation Act 1999. Blood samples were collected under Department of Environment and Primary Industry permits (10005392 and 10007008 under the Wildlife Act 1975 and the National Parks Act 1975) and Office of Environment and Heritage New South Wales permit (100850 under the National Parks and Wildlife Act 1974), and approval of animal ethics committees of Victorian Department of Environment and Primary Industry (10/33 and 14/07), New South Wales Department of Environment and Primary Industry (10/5601), and Taronga Conservation Society Australia (R12L129).

**Table 1:** Geographic locations of sampling sites, sample sizes for each individual location, and pooled sample sizes. The birds at Sutton were pooled with those from Canberra, birds from Indigo Valley and Lurg were pooled with those from Chiltern, and birds captured at Cumbo Rd, Goulburn River, and Munghorn Gap were pooled together under the name Goulburn River for analyses.

Location	Latitude	Longitude	Number of samples captured at each site	Number of samples pooled for analyses
Armidale	30.516756 S	151.66671 E	23	23
Canberra	35.300451 S	149.133313 E	7	9
Capertee	33.149836 S	149.982577 E	35	35
Chiltern	36.149813 S	146.599959 E	15	17
Cumbo Rd, NSW	32.375377 S	149.893828 E	3	-
Goulburn River NP	32.272195 S	149.858295 E	2	6
Indigo Valley	36.182488 S	146.698728 E	1	-
Lurg	36.585148 S	146.117383 E	1	-
Munghorn Gap	32.389558 S	149.82297 E	1	-
Quorrobolong	32.920179 S	151.333699 E	9	9
Sutton	35.166164 S	149.249966 E	2	-

### Laboratory Methods

DNA extraction, microsatellite amplification for 16 microsatellite loci, and molecular sexing using CHD primers P2 and P8 (S2 Table) were completed following Harrisson *et al*. (2014a) [29]. Quality control included randomly rescreening 10% of the samples.

### Validation of appropriate behavior of genetic markers

Most rescreening disagreements were with locus BMC4; this locus was removed, reducing the error-rate from 1.30% to 0.20% per individual-allele combination. A total of 15 loci were included in analyses, 10 of which were polymorphic. Loci were tested for Z-linkage by checking for the presence of heterozygotes and homozygotes of both sexes. Detection of null alleles was carried out by examination of the data for homozygous nulls (i.e. individuals who fail to amplify at a locus during multiplex reactions despite other loci amplifying) and consistent patterns of homozygous excess at capture sites using GENALEX 6.501 [41–42]. *F*_IS_ was calculated for each locus using GENEPOP 4.2 [43–44]. To examine possible Wahlund effect, *F*_IS_ was calculated for each locus and Hardy-Weinberg equilibrium tested, treating each sampling site as a separate population. To avoid discarding loci with sufficient variability to detect patterns and maximize sample size, while at the same time accounting for possible null alleles found at locus Pn1, analyses were performed using all loci (except BMC4) and omitting locus Pn1. The null allele frequency for Pn1 was calculated using Brookfield equation 1 in MICRO-CHECKER, as non-amplification may have been the result of either null alleles or issues with DNA [45–46].

### Patterns of genetic diversity

For analyses, wild birds sampled at sites with fewer than five individuals were pooled with the geographically next closest site; the maximum distance between any two sites where birds were pooled together was 69 km, and six sites were analyzed overall (Fig 1). Birds at geographically nearby sites were tested for significantly different allele frequencies using genic differentiation in GENEPOP 4.2 in order to determine if they could be pooled together for analyses. To examine patterns of allelic diversity, allelic richness was calculated in FSTAT 2.9.3 and standardized using rarefaction for birds sampled at each site (minimum sample size of 4 individuals), wild birds sampled before 2000 and after 2010 as no birds were sampled between these years (minimum sample size of 27 individuals), and for the wild and captive populations (minimum sample size of 83 individuals) [47]. Because absence of alleles may indicate either extinction or sampling error, the Wilcoxon paired samples signed rank test was used to test for significance of the difference in allelic richness in wild birds captured before 2000 and after 2010. To determine if similar allele frequencies occurred at different sites, and how many alleles may have become extinct over the course of sampling, allele frequencies for each site and for wild birds captured before 2000 and after 2010 were calculated in GENALEX; private alleles were also calculated in GENALEX for wild birds captured before 2000 and after 2010. As an additional insight into genetic variation, mean observed and mean expected heterozygosity was calculated in GENALEX for wild birds sampled before 2000 and after 2010, and for the wild and captive populations.

### Patterns of genetic differentiation

To measure the allelic differentiation between wild-caught regent honeyeaters at different sites and between the wild birds and founders of the captive populations (information taken from pedigree charts), pairwise *F*_ST_ values were calculated and tested for significance in GENEPOP 4.2. To determine if the amount of differentiation in wild birds had changed over time, average *F*_ST_ over sampled locations was calculated for wild birds sampled before 2000 and after 2010 in AMOVA in GENALEX 6.501; 999 permutations were used, individuals with data missing at more than two loci were removed, and remaining missing data points were interpolated. Isolation-by-distance was tested with Mantel tests in GENALEX 6.501. Mantel tests between *F*_ST_ or linearized *F*_ST_ and geographic distance or log(1+geographic distance) were performed using 999 permutations. Birds with data missing at two or more loci were not included in Mantel test calculations, and remaining missing data were interpolated by GENALEX as missing data may be problematic in pairwise, distance based analyses.

Genotypic differentiation in wild adults, wild birds sampled before 2000, and wild birds sampled after 2010 was tested in STRUCTURE 2.3.4, completed with a burn-in length of 1 x 10^6^ and 3 x 10^6^ replications, setting K from 1 to 5, and performing 10 iterations for each K [48]. STRUCTURE HARVESTER v0.6.94 was used to determine the K with the highest lnP(D), as the Evanno *et al*. (2005) method is not appropriate when there is a realistic chance the true number of genotypic clusters could be one [49–50]. Evidence of geographic structure was tested for wild birds in TESS 2.3 assuming 2–5 clusters (K), and using the BYM model with spatial interaction parameters P = 0.6, trend degree T = 2, and D = 1.0 for the Dirichlet allele frequency model [51–52]. A total of one hundred replicates of 3 x 10^4^ burn-in sweeps followed by 1 x 10^5^ sweeps were run per K. Individuals with data missing at more than two loci were excluded from analyses. TESS GUI was used to find the 10 runs with the lowest DIC value for each K, and cluster probabilities were averaged together to estimate the true K [51].

### Philopatry and mobility within the species and each sex

Sex differences in philopatry and mobility are expected to lead to the more philopatric sex having stronger spatial genetic patterns than the more dispersive sex [53]. To test for sex-biased dispersal, as well as genetic patterns within the species, spatial autocorrelation analysis was performed using GENALEX 6.501 for adult males and females of wild-caught regent honeyeaters. Analyses were performed for each sex separately as well as both sexes together. Bin sizes were chosen to represent distances within and between breeding sites. Birds with missing data at two or more loci were removed from the dataset, and any remaining missing data were interpolated. The heterogeneity test of Smouse *et al*. (2008) was used to test the significance of differences, and significance was declared at p < 0.01 following Banks and Peakall (2012) [54, 53]. All analyses used 999 random permutations, 95% confidence intervals around r, and 999 bootstraps to estimate confidence intervals.

### Degrees of relatedness among sites and within the captive population

To test for geographically different levels of relatedness of birds, mean within-population pairwise R-values were calculated for wild birds sampled at different sites, as well as for the captive population. As a calibration for interpreting mean within-population pairwise R-values in the wild population, distributions of pairwise R-values were calculated for the 90 captive birds for parent-offspring pairs, full-sibs, half-sibs, and unrelated birds using pedigree data in GENALEX 6.501 following Queller and Goodnight (1989) [55]. To test if high mean within-population pairwise R-values were due to samples including families, CERVUS 3.0 was used to find parent-offspring pairs within the data, with confidence intervals calculated using LOD scores [56].

### Population Sizes

To test if regent honeyeaters have undergone a recent genetic bottleneck, BOTTLENECK 1.2.02 was used to calculate the probability of heterozygosity excess (indicative of a recent bottleneck) or deficit, relative to that expected from the number of alleles present, in birds captured in the wild before 2000 and after 2010 [57]. This was done by comparing measured heterozygosity values and heterozygosity values at mutation-drift equilibrium for each locus, and by using Wilcoxon tests using the two-phase model, as it is a better estimator than the stepwise mutation model [58]. The default parameters for the two-phase model were used, with variance set to 30 and the proportion of single mutation model in the two-phase model set to 70%. Effective population sizes for the pre-2000 and post-2010 wild populations, as well as for each site, were calculated using ONeSAMP 1.2; 50000 iterations were used, generation size was set from 4–1000, and individuals missing genotypes at two or more loci were removed [59]. Because using one sample of genotypes to estimate effective population size may produce different values than an estimation based off of the amount of genetic drift between samples taken at different time periods, contemporary effective population size was also calculated in NeEstimator 2.01 [60]. Plan I of the temporal method was used as it assumes individuals were non-lethally sampled; census sizes of 1500 and 400 were assumed for birds sampled prior to 2000 and after 2010 respectively [19, 27]. A generation time of one year was used as birds begin to reproduce at one year, and the minimum allele frequency was set to 0.01 [27]. Effective population size, as well as a measure of the standard variance of genetic drift between temporal samples, *F*_C_, was calculated using the methods of Nei and Tajima (1981) [61].

### Ability of data to detect genetic differentiation

To determine the power of the data to detect patterns of differentiation, simulations were conducted using EASYPOP 2.0.1 to model habitat fragmentation isolating the species into two demes for varying numbers of generations and effective population sizes [62]. Simulations were performed with varying levels of migration (0, 0.1, and 0.01) for 150 generations (time since onset of major habitat fragmentation, as regent honeyeaters reproduce after one year), 40 generations (time since regent honeyeater decline was first observed), and 10 generations (gap in sampling between 2000 and 2010) [27]. An effective population size of 400 birds was chosen to represent the estimated population size of at least 1500 birds until at least the 1990s, and an effective population size of 100 birds was chosen to represent the current estimated population size of 400 birds [19, 27]. These effective population sizes were consistent with our estimates (see results). All simulations were completed under the parameters of complete monogamy, 10 loci of six alleles each, populations of equal size, equal numbers of females and males in each population, and equal migration rates between females and males. A total of 10 runs were performed for each scheme of effective population size and number of generations. To examine the effects of small sample size on the ability of the data to detect differentiation, the mean, range, and standard deviations of *F*_ST_, as well as p-values, were calculated for the first 10, 25, 50, and 200 (for effective population sizes of 400 only) individuals from each deme by running simulation results in GENEPOP 4.2.

## Results

### Validation of appropriate behavior of genetic markers

A total of 189 regent honeyeaters (108 wild-caught and 81 captive) were genotyped at 15 loci, 10 of which were polymorphic. Although sample sizes were small, they include a substantial proportion of the regent honeyeater population; small sample size is an unavoidable consequence of studying endangered species. There was limited success of reliable screening of feather samples, and so these were excluded from analyses. No loci were sex-linked. Two loci (BMC2 and Pn1) showed significant homozygous excess when the birds were treated as one population (S3A Table), indicating possible Wahlund effect or null alleles. This pattern was maintained for Pn1 but not BMC2 for all sites when samples were treated as six populations based on capture site (S3B Table), suggesting that Pn1 but not BMC2 had null alleles. Congruently, three wild-caught birds had putative homozygous nulls at Pn1 (repeated non-amplification of otherwise good DNAs), but there were no such candidates for BMC2. MICRO-CHECKER estimated the null frequency of Pn1 to be 0.0973 using Brookfield equation 1 [46, 45]. The presence of null alleles does not necessarily impact the outcomes of population genetic analyses: their presence tends not to have large consequence in analyses that use average probabilities (as opposed to individual parentage analyses), but they may cause overestimation of *F*_ST_ and genetic distances, and slightly lower the power of assignment tests (such as STRUCTURE) [63–65]. Omitting locus Pn1 returned similar results as including all loci for analyses (S1 Results), and so results are reported including Pn1.

### Weak population genetic differentiation within the species

**Fig 2:**
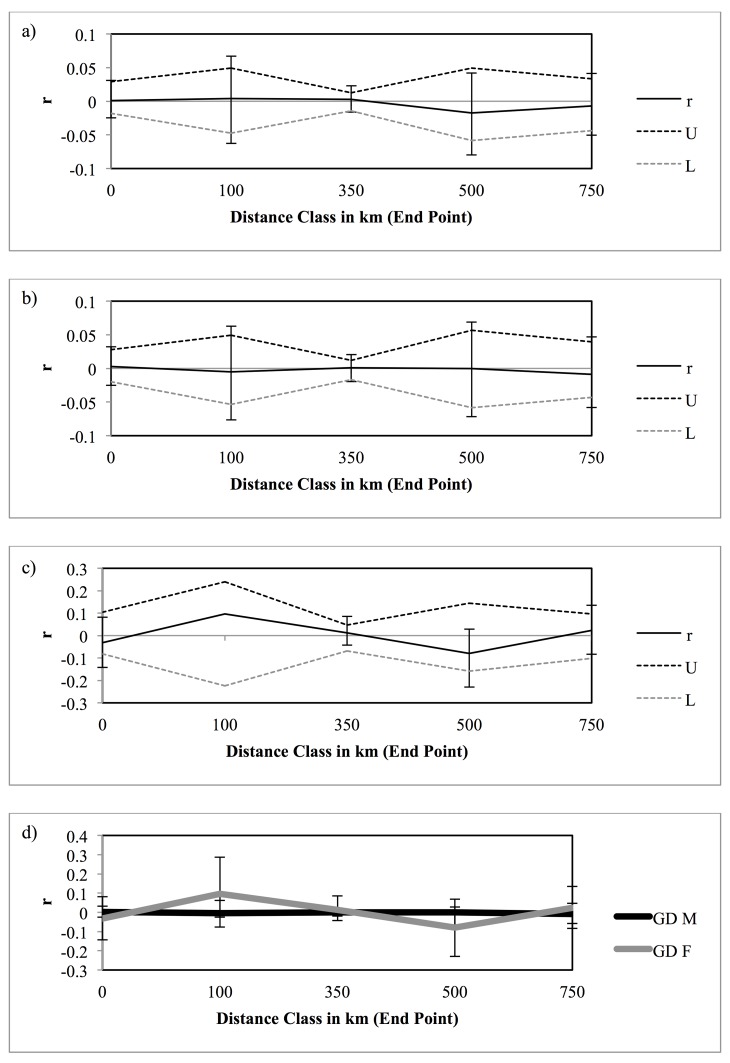
Spatial autocorrelation analysis showing spatial genetic structure in a) adult regent honeyeaters; b) adult males; c) adult females; and d) differences in structure between each sex. No distance classes had significant (p < 0.01) r-values for any group, and no distance classes had r-values that were significantly different (p < 0.01) between males and females.

There were no significant differences in allele frequencies between populations that were pooled together for analyses (p = 0.3605–0.9899). Similar allelic diversity occurred across sites (S4 and S5 Tables). Mean allelic richness was slightly lower in captive birds than in wild birds, but the opposite was true for heterozygosity (S6 Table). There was little change in mean observed and expected heterozygosity for wild birds sampled before 2000 or after 2010 (S7 Table). There are 15 alleles that occur in the pre-2000 samples but not in the post-2010 samples. However, this may be due to sampling error rather than allele loss: 6 alleles occur in the post-2010 samples that are not seen in the pre-2000 samples, there are three times as many pre-2000 samples (81 birds) as there are post-2010 (27 birds), and little change in allelic richness (which does control for sample size) between the two groups (p = 0.509) (S7 and S8 Tables). Similarly, there was little evidence of genetic differentiation between wild-caught regent honeyeaters from different sites (Table 2): pairwise *F*_ST_ values were low (0–0.0254) for all site comparisons, and most were not significant. There was small but significant differentiation between wild-bred and captive-bred samples (*F*_ST_ = 0.0296, p = 0.018). Differentiation in wild birds was found by AMOVA to be similar throughout sampling periods, with *F*_ST_ = 0.002 (p = 0.393) for wild birds sampled before 2000, and *F*_ST_ = 0.003 (p = 0.402) for wild birds sampled after 2010. No genotypic differentiation was found by STRUCTURE within the subsets of wild-only adult birds, birds sampled before 2000, or birds sampled after 2010 (K = 1 was the most probable number of genotypic clusters for each subset). Mantel tests showed no significant relationship between genetic and geographic distance, with or without transformation (Rxy ranged from -0.189 –-0.004, p from 0.252–0.424, S1 Fig). The K = 2 analysis in TESS assigned all individuals to the same cluster so K = 1 was inferred. Spatial autocorrelation found no spatial genetic patterns within the species as a whole or in either sex (Fig 2). While having only six sampling locations dictates that tests for isolation-by-distance (Mantel tests) will be underpowered, the results of no spatial structure correspond well with the STRUCTURE results of K = 1, as well as the lack of structure in the individual-based spatial autocorrelation results (Fig 2A); for this reason, isolation-by-distance tests are included here for completeness.

**Table 2:** Pairwise F_ST_ values for wild-caught regent honeyeaters at different sites.

Site	Armidale	Canberra	Capertee	Chiltern	Goulburn River
**Canberra**	0.0001				
**Capertee**	0.0071***	0			
**Chiltern**	0.0188***	0	0		
**Goulburn River**	0.0008	0.0026	0.0154*	0.0105	
**Quorrobolong**	0.0234***	0	0.0022	0	0.0254

Asterisks indicate significance

* = p < 0.05

** = p < 0.01

*** = p < 0.001.

### Differential degrees of relatedness across sites

**Fig 3:**
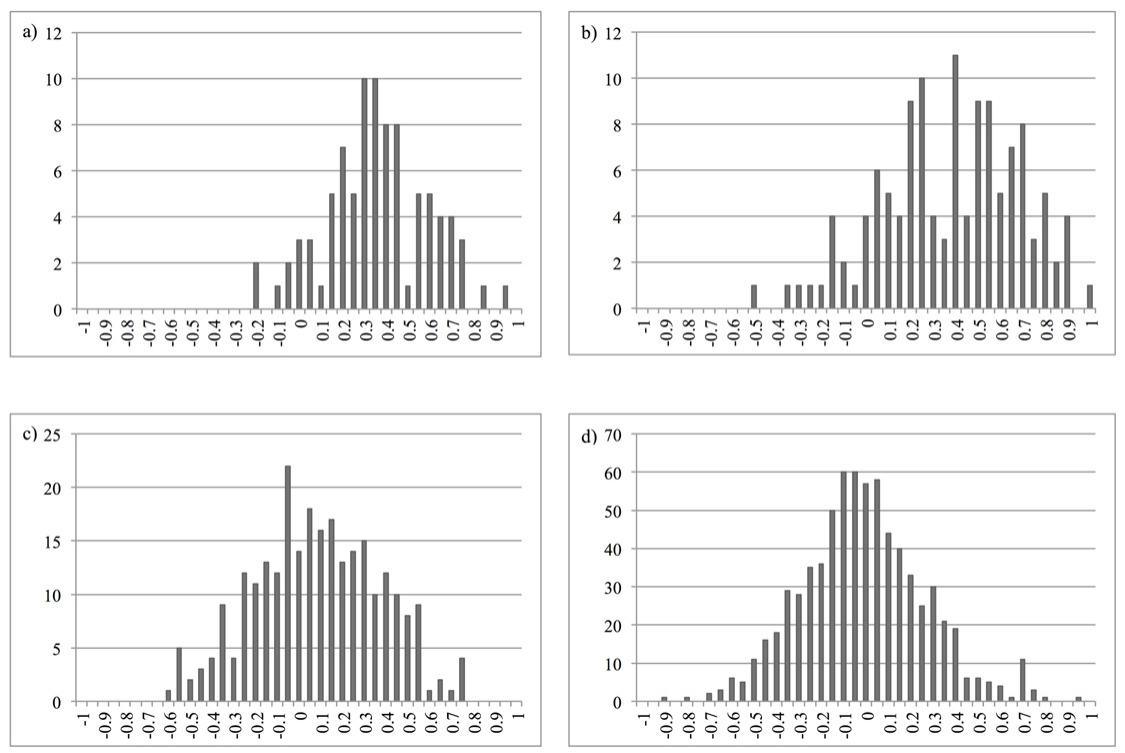
R-value distributions of regent honeyeaters of known pedigree at Taronga Zoo for a) parents and offspring (mean R-value 0.3367); b) full-sibs (mean R-value 0.3498); c) half-sibs (mean R-value 0.0578); and d) unrelated birds (mean R-value -0.0369).

**Fig 4:**
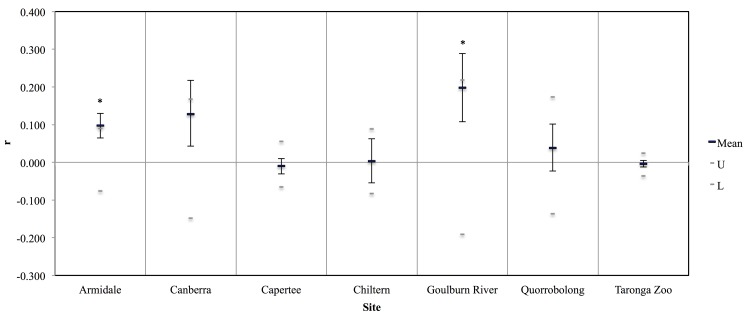
Mean r-values for birds at each site. Wild-bred founders of the captive population and captive-bred birds released into the wild were included in calculations as members of both Taronga Zoo and the wild site at which they were (re)captured. An asterisk (*) indicates an r-value is significantly (p < 0.05) different from zero.

Captive birds of known pedigree had normal R-value distributions, shifted lower than theoretical expectations (Fig 3); this shift may reflect active genetic management (inbreeding avoidance) of the captive population. The differences in distributions of R among the different levels of pedigree relatedness indicate that the genetic assay applied here provides some meaningful signal of genotypic similarities. Mean R-values ranged from -0.010 to 0.198 for wild-caught birds within the sites at which they were captured (Fig 4). Using the mean R-values for the captive birds as a calibration indicates that mean relatedness among birds at Armidale and Goulburn River exceeds that of half-sibs, while the wild birds are on average randomly related at other sites; however, overlapping 95% confidence intervals for all sites suggest there is no significant difference between sites. There was not enough variability within the loci for CERVUS to return consistent parentage results among independent runs of the program.

### Small and declining population size

During a population bottleneck, the number of alleles is expected to decline faster than heterozygosity, leading to an excess of heterozygosity than what is expected for the observed number of alleles [57]. Despite a clear, recent demographic bottleneck, BOTTLENECK results revealed no evidence for a recent genetic bottleneck (S9A Table). Rather than the heterozygous excess relative to mutation-drift equilibrium that can characterize genetic bottlenecks, there was heterozygous deficit at nearly all loci (S9B Table). The effective population size was estimated to have undergone a significant decline between 2000 and 2010, and all sites (except Goulburn River, which had sample sizes too low for effective population size estimation by ONeSAMP) were estimated to have low (13–98) effective population sizes (Table 3). NeEstimator estimated the contemporary effective population size to be 149 (95% parametric CIs = 68–147). The standard variance of allele frequency changes, *F*_C_, was calculated to be 0.04544 between pre-2000 and post-2010 samples.

**Table 3:** Estimated mean effective population sizes as estimated by ONeSAMP for different sampling locations and for wild birds sampled before 2000 and after 2010. Sample sizes at Goulburn River were too small for effective population size estimation.

Sample	Effective Population Size	95% CL
**Armidale**	47	34–118
**Canberra**	16	13–25
**Capertee**	98	67–281
**Chiltern**	18	14–33
**Quorrobolong**	13	11–19
** **		
**Pre-2000 Wild Birds**	673	317–3347
**Post-2010 Wild Birds**	87	61–226

### The present data would have sufficient power to detect low levels of migration

Simulations from EASYPOP 2.0.1 showed the data to have power to detect genetic differentiation through *F*_ST_ for all tested migration rates when effective populations size was set to 100, and low (0–0.1) rates of migration when effective population size was set to 400 (S10 Table). While similar patterns of genetic differentiation were detected for all sample sizes, the number of significant *F*_ST_ values decreased with smaller sample size. This was especially true at high rates of migration, greater effective population size, and higher numbers of generations.

## Discussion

Regent honeyeaters exhibit little population genetic differentiation between birds captured at different sites. The low structure was consistent over time in our samples: birds sampled before 2000 and after 2010 were equally unstructured. There were also no significant differences in mobility between males and females. The genetic similarity of birds across all locations sampled suggests the wild population is close to a single genetic unit, with gene flow occurring approximately species-wide, consistent with prior knowledge of the high mobility of the species [16].

### Low levels of differentiation across sites concurrent with localized relatedness

Little evidence of genetic differentiation in the wild population (Table 2, Fig 2), as well as no pattern of isolation-by-distance, indicates that the wild population of regent honeyeaters is close to panmictic. Although four pairwise *F*_ST_ values for wild birds captured at different sites were statistically significant, all but one are less than 0.02 (one is very slightly higher); this was defined by Lowe and Allendorf (2010) to be the threshold for drift connectivity whereby similar allele frequencies in subpopulations are maintained by gene flow [3]. High levels of gene flow within a species do not, however, preclude local structure from occurring [66–67, 29]. Low levels of allele frequency differentiation throughout the species are not in conflict with high average genotypic relatedness at some sites (Fig 4) owing to local family structure following breeding. Also, families may move and feed together; Franklin *et al*. (1989) suggest the movements of regent honeyeaters are not random, but instead may be learned from other individuals [16]. Low variability within the data prevented confirmation of family structure. Cooke and Munro (2000) found evidence of genetically controlled seasonal movements, which may also explain high average relatedness at some sites [68].

Because low levels of genetic differentiation implies gene flow is occurring at the landscape level, habitat fragmentation does not appear to have severely limited the movements of the regent honeyeater. However, the effects of habitat loss and fragmentation on a species are not always immediately apparent in genotype and allele frequencies, and may only become apparent after many generations [69]. Although enough time has passed both since the initial habitat loss and the later demographic decline of the species for genetic differentiation (measured by *F*_ST_) to occur under low levels of migration (S10 Table), a mobile species such as the regent honeyeater may have migration rates too high for genetic drift to allow significant differentiation to accumulate. While it does not appear that habitat fragmentation has entirely isolated regent honeyeaters at any particular site, it may have limited migration enough to cause a higher degree of relatedness of birds at some sites than others. Such differential relatedness across sites may be an early indication, not yet widely observable in other tests, of some restriction of gene flow. Furthermore, resightings of wild regent honeyeaters have shown birds moving within and between all sites from where they were banded, in some cases demonstrating long-distance (up to 580 km) movements of individuals that would facilitate low levels of differentiation [70].

### Lack of temporal change in genetic diversity

Because no signal of genetic clustering could be detected by STRUCTURE for wild birds sampled before 2000 or after 2010, and because AMOVA did not find significant differentiation in either group, it appears that the low differentiation within the species has been temporally continuous. This suggests that habitat fragmentation has neither triggered novel population structure by limiting dispersal nor erased historic structure by forcing birds to disperse to habitat patches to which they would not normally travel, over the timescale encompassed by the sampling. However, it is important to note that all sampling was subsequent to the demographic decline; had more temporally extensive sampling been possible, pronounced temporal changes in genetic diversity may potentially have been observed.

Lack of continued loss of alleles (S7 and S8 Tables) despite effective population declining over the course of sampling (Table 3), as well as a low *F*_C_ value between pre-2000 and post-2010 samples, suggests the continued mobility of this species may be offering some buffer against genetic drift; this may have dampened bottlenecking effects enough that they were not so extreme on the timescale under consideration to be detected by the BOTTLENECK test with the present data. There are many possibilities for why a genetic bottleneck might not be detectable, including uncertainties about mutation models of the loci, and population structure and gene flow through time. For example, habitat loss and population contractions may have increased gene flow through enforced mobility to obtain resources, and bringing in additional rare alleles can mask or reverse heterozygosity excess [57].

### Mobility and sex differences in philopatry

Many highly mobile species exhibit strong population structure as a result of natal philopatry, and greater population structure is expected in the more philopatric or less mobile sex [71, 67]. Although some site fidelity has previously been noted in regent honeyeaters, including that specific to males, spatial autocorrelation did not support these observations (Fig 2); as such, strong sex-bias in dispersal does not appear to be part of the biology of this species [37]. That little spatial structure was found in either sex supports observations that regent honeyeaters must exhibit high mobility as a means of obtaining nectar; such movements may result in opportunistic breeding at feeding grounds, resulting in gene flow across the landscape [16].

### Genetic differences between captive and wild birds

Little differentiation between the captive and wild regent honeyeater populations indicates the species may be managed as one unit, without concern for whether progeny of wild birds and captive releases would be at particular risk of either inbreeding- or outbreeding depression. While *F*_ST_ indicates there is small but significant differentiation between captive and wild samples, this may be due to stochasticity; the founders of the captive population were taken from the wild, and the low substructure in the wild birds suggests they would not be strongly differentiated from the rest of the wild population. Similar levels of genetic diversity within the wild and between the wild and captive populations (S6 Table, S4 Table) indicate that the captive population is currently a reasonable neutral genetic representation of the wild population.

### Implications for conservation and management

Because there is evidence of only minor genetic structure in this species, the low genetic diversity found in this study is likely to be occurring throughout the species range; it is unlikely that there are unsampled pockets of substantial genetic diversity remaining in the wild. Low population genetic diversity, as is expected following severe demographic decline, has potentially serious implications for the future of species in the situation faced by regent honeyeaters. The major genetic threats are inbreeding depression, and the reduction of evolutionary potential that may lessen the ability of a species to adapt to future environmental change [72–74]. The low levels of differentiation within and mobility of this species may be of benefit as it maximizes the number of potential mates, particularly if long-distance dispersers do not experience much reduced fitness, as occurs in some species [75]. However, if the high degree of relatedness in birds at some sites is due to recently reduced gene flow, inbreeding could become more of a threat. However, as indicated above, there are no compelling reasons to infer timelags: regent honeyeaters have low population structure and may merely show minor transient local structure, as do many species of bird. Even under panmixia, it is inescapable that small populations lose genetic diversity over time.

Recent considerations suggest that an effective population size of >100 individuals is required to limit loss in total fitness to <10% over five generations, and retaining evolutionary potential in perpetuity requires >1000 effective individuals; as the current effective population size is estimated to be only between 87 and 149 birds depending on calculation method, regent honeyeaters are at risk of inbreeding depression and very high risk of loss of evolutionary potential [76]. However, the estimate of an effective population size of 149, while small, may include some error and be an overestimation. This is due to large demographic changes occurring at a variable rate over the course of sampling, and because the temporal method of effective population size estimation does not take iteroparity (as is the regent honeyeater) into account; however, detailed data of age structure and birth rates that may allow for more accurate effective population size estimation are not available for this species [77–78, 61]. Despite this, adequate sampling over enough generations is expected to lead to estimates converging on the true effective population size, and overlapping generations should not cause major error [77–78, 61]. Because the typical N_e_/N ratio for birds is 0.21, a current population of 400 should have an effective population size of around 84; the ONeSAMP estimate of 89 is much closer to this value than the NeEstimator estimate of 149 [79, 19]. While an effective population size of 149 would indicate regent honeyeaters faced somewhat less risk of inbreeding depression than one of 84–89, 149 may be an overestimate. However, as all estimates are very small, continued efforts to restore habitat and maximize effective population size are extremely important.

The genetic similarity between the captive and wild bird populations suggests low potential for genetic rescue by captive release into the wild [80]. Nonetheless, as long as genetic and physical health of the captive population is maintained, continued release of regent honeyeaters into the wild can provide demographic benefits. This species must often compete for access to nectar sources with larger, more aggressive birds such as Noisy Miners *Manorina melanocephala*, Red Wattlebirds *Anthochaera carunculata*, and Noisy Friarbirds *Philemon corniculatus*; aggregate nesting may allow regent honeyeaters to exhibit group defense against competitive species, but population decline threatens their ability to engage in this behavior [16, 18, 81]. Captive release would contribute to higher numbers in the wild, allow for larger breeding congregations, and increase interspecific competitiveness.

Because there is little genetic structure in regent honeyeaters, it is unlikely that genetic subdivision will be of concern for the release of captive birds; the wild and captive populations may be managed together, and effective population size maximized by attention to the relatedness of breeders [82].

Because current microsatellites for regent honeyeaters have low diversity and sample sizes are small, they have somewhat limited statistical power for monitoring this species. Therefore, the future development of genomic techniques that yield hundreds or thousands of loci is worthwhile to achieve specific monitoring goals, such as breeding between captive-bred and wild birds, and tracking genome-wide diversity for its implications for evolutionary potential [73]. Sampling should continue so that highly resolving genetic assays may be used to track individual reproductive success through parentage analyses, and to better detect declines in genetic variation over long time periods.

## Supporting Information

S1 Data Genotype data used in analyses. (XLSX)

S1 Fig Plots of geographic distance vs genetic differentiation in wild birds for a) geographic distance vs *F*_ST_; b) geographic distance vs linearized *F*_ST_; c) log(1 + geographic distance) vs *F*_ST_; and d) log(1 + geographic distance) vs linearized *F*_ST_. (DOCX)

S1 Results Analyses omitting locus Pn1. (DOCX)

S1 Table List of individual samples, year sampled, sex (U indicates unknown), and location sampled. A cross (†) indicates the individual was an adult at the time of sampling. One asterisk (*) indicates the bird was a captive-bred bird released from Taronga Zoo, and two asterisks (**) indicate the bird was a wild-bred founder of the captive population.
(DOCX)

S2 Table List of microsatellite loci (with sources) and primers used in multiplex reactions. (DOCX)

S3 Table List of 15 loci used in analyses and the number of alleles, allele size, probability of significance for Hardy-Weinberg tests (only significant results shown), and *F*_IS_ values. Table a includes all wild and captive birds (N = 189) in calculations for the number and size of alleles; Hardy-Weinberg and *F*_IS_ values are calculated using only wild birds (N = 108) treated as one population. Table b shows the probability of significance of Hardy-Weinberg tests (only significant results shown) and *F*_IS_ values when birds are treated as six populations based on where they were captured. Locus HrU2 did not have enough allelic diversity to calculate *F*_IS_ at Canberra (monomorphic except for one allele in 1 individual).
(DOCX)

S4 Table Allele frequencies for each locus by site. (DOCX)

S5 Table Allelic richness (AR) by geographic location for each polymorphic locus. Values in parentheses are standard errors.
(DOCX)

S6 Table Allelic richness (AR) and observed (H_O_) and expected (H_E_) heterozygosities for polymorphic loci in the wild and captive populations. Values in parentheses are standard errors.
(DOCX)

S7 Table Allelic richness (AR) and heterozygosity for wild regent honeyeaters sampled before 2000 and after 2010. N is the number of sampled individuals in the time period, H_O_ is the observed heterozygosity, H_E_ is the expected heterozygosity, and No private alleles refers to the number of alleles found only in samples from one time period. Note this analysis includes only polymorphic loci. Standard errors are given in parentheses.
(DOCX)

S8 Table List of allele frequencies for each polymorphic locus for wild-bred birds sampled pre-2000 and post-2010. Alleles with a frequency of zero in one of the samples are shaded gray; such alleles may represent a sampling issue or, if not found in the post-2010 sample, extinction.
(DOCX)

S9 Table Estimated heterozygosity excess or deficit as calculated by the two-phase model in BOTTLENECK for regent honeyeaters captured in the wild for a) all loci together using the Wilcoxon test and b) measured heterozygosity (H_e_) and heterozygosity at mutation-drift equilibrium (H_eq_) for each polymorphic locus. An excess indicates a recent population size bottleneck.
(DOCX)

S10 Table EASYPOP 2.0.1 simulations demonstrating the ability of the data to detect changes in genetic differentiation for several schemes of migration rate, generation time, and effective population size for a sample size of a) 10 birds per deme; b) 25 birds per deme; c) 50 birds per deme; and d) 200 birds per deme (for an effective population size of 400 only). The mean, range, and standard deviation (SD) for *F*_ST_ represent that of 10 runs, and the number of those runs with significant *F*_ST_ values is shown.
(DOCX)
