# Supplementary material for: Very Low Population Structure in a Highly Mobile and Wide-Ranging Endangered Bird Species
Source: PLoS One. 2015 Dec 9;10(12):e0143746. doi: 10.1371/journal.pone.0143746 (PMC4674126; doi:10.1371/journal.pone.0143746)
Supplement: S1 Fig — (DOCX) [file pone.0143746.s002.docx]

**S1 Figure: Plots of geographic distance vs genetic differentiation in wild birds for a) geographic distance vs *F*_ST_; b) geographic distance vs linearized *F*_ST_; c) log(1 + geographic distance) vs *F*_ST_; and d) log(1 + geographic distance) vs linearized *F*_ST_.**

a)

b)

c)

d)
