# Supplementary material for: Very Low Population Structure in a Highly Mobile and Wide-Ranging Endangered Bird Species
Source: PLoS One. 2015 Dec 9;10(12):e0143746. doi: 10.1371/journal.pone.0143746 (PMC4674126; doi:10.1371/journal.pone.0143746)
Supplement: S1 Results — (DOCX) [file pone.0143746.s003.docx]

**S1 Results: Analyses omitting locus Pn1.**

**Allelic differentiation by pairwise *F*_ST_ values**

**Table 1: Pairwise *F*_ST_ values for wild-caught regent honeyeaters at different sites as calculated in GENEPOP 4.2.** One asterisk (*) indicates p < 0.05. No values had significance of p < 0.01 or 0.001:

| **Site** | **Armidale** | **Canberra** | **Capertee** | **Chiltern** | **Goulburn River** |
| --- | --- | --- | --- | --- | --- |
| **Canberra** | 0.0007 |  |  |  |  |
| **Capertee** | 0 | 0 |  |  |  |
| **Chiltern** | 0.0097* | 0 | 0.0025 |  |  |
| **Goulburn River** | 0.0055 | 0.0134 | 0.0146 | 0.0076 |  |
| **Quorrobolong** | 0.0104 | 0 | 0.0054 | 0 | 0.0210 |

The pairwise *F*_ST_ value for the wild birds and breeders within the captive populations is 0.0278, p < 0.001. *F*_ST_ values calculated by AMOVA were 0.002 (p = 0.333) for wild birds sampled before 2000, and 0.007 (p = 0.305) for wild birds sampled after 2010.

**Isolation-by-distance**

Mantel tests revealed no relationship between *F*_ST_ or linearized *F*_ST_ and geographic distance or log(1+geographic distance) (Rxy ranged from -0.189 – 0.002, p from 0.243 – 0.419).

**Fig 1: Plots of geographic distance vs genetic differentiation in wild birds for a) geographic distance vs *F*_ST_; b) geographic distance vs linearized *F*_ST_; c) log(1 + geographic distance) vs *F*_ST_; and d) log(1 + geographic distance) vs linearized *F*_ST_.**

a)

b)

c)

d)

**Genotypic differentiation**

STRUCTURE and TESS analyses were not repeated without Pn1 as excluding Pn1 from pairwise differentiation analyses caused pairwise-*F*_ST_ values to decrease; Harrisson et al. (2012) showed that pairwise differentiation is more sensitive than STRUCTURE, and the STRUCTURE results for all loci did not find any genotypic differentiation within the species [66].

**Spatial Autocorrelation**

**Fig 2: Spatial genetic structure in a) adult regent honeyeaters; b) adult males; c) adult females; and d) comparison of structure between adult males and adult females.** No r-values were significant (p < 0.01) at any distance class, and spatial structure in males did not differ significantly (p < 0.01) from spatial structure in females at any distance class.

a)

b)

c)

d)

**Mean Within Population Pairwise R-values**

**Fig 3: Mean within population pairwise R values following Queller and Goodnight (1989) [55].** Sites with an asterisk (*) indicate statistically significant values (p < 0.05).


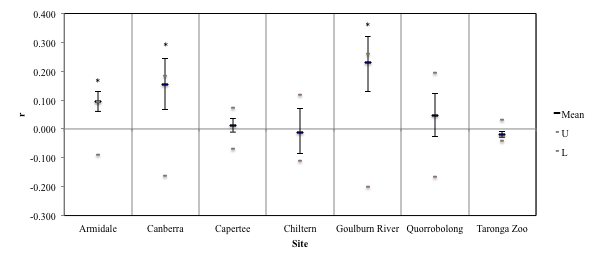


Matrices of R-values within the captive population following Queller and Goodnight (1989) for all polymorphic loci and for polymorphic loci excluding Pn1 were highly correlated in a Mantel test in GENALEX (Rxy = 0.930, p < 0.001) [55].

**Bottlenecking**

**Table 2: Estimated heterozygosity excess or deficit as calculated by the two-phase model in BOTTLENECK for regent honeyeaters captured in the wild using the Wilcoxon test.**

| **Pre-2000 Samples** |  |
| --- | --- |
| Probability (one tail for heterozygosity deficiency) | 0.082 |
| Probability (one tail for heterozygosity excess) | 0.936 |
| Probability (two tails for heterozygosity excess and deficiency) | 0.164 |
| **Post-2010 Samples** |  |
| Probability (one tail for heterozygosity deficiency) | 0.064 |
| Probability (one tail for heterozygosity excess) | 0.976 |
| Probability (two tails for heterozygosity excess and deficiency) | 0.129 |

**Effective Population Size**

Removal of Pn1 left insufficient data for NeEstimator to accurately estimate effective population size from temporal changes in genetic drift (effective population size was estimated to be several times higher than the census size), so this is excluded from analyses.

**Table 3: Estimated mean effective population sizes as estimated by ONeSAMP for different sampling locations and for wild birds sampled before 2000 and after 2010.** Sample sizes at Goulburn River were too small for effective population size estimation.

| **Sample** | **Effective Population Size** | **95% CI** |
| --- | --- | --- |
| **Armidale** | 40 | 27 - 113 |
| **Canberra** | 10 | 9 - 17 |
| **Capertee** | 132 | 84 - 371 |
| **Chiltern** | 17 | 13 - 32 |
| **Quorrobolong** | 12 | 10 - 20 |
| **Pre-2000 Wild Birds** | 191 | 106 - 569 |
| **Post-2010 Wild Birds** | 88 | 58 - 257 |
