# Supplementary material for: Very Low Population Structure in a Highly Mobile and Wide-Ranging Endangered Bird Species
Source: PLoS One. 2015 Dec 9;10(12):e0143746. doi: 10.1371/journal.pone.0143746 (PMC4674126; doi:10.1371/journal.pone.0143746)
Supplement: S1 Table — A cross (†) indicates the individual was an adult at the time of sampling. One asterisk (*) indicates the bird was a captive-bred bird released from Taronga Zoo, and two asterisks (**) indicate the bird was a wild-bred founder of the captive population. (DOCX) [file pone.0143746.s004.docx]

**S1 Table: List of individual samples, year sampled, sex (U indicates unknown), and location sampled.** A cross (†) indicates the individual was an adult at the time of sampling. One asterisk (*) indicates the bird was a captive-bred bird released from Taronga Zoo, and two asterisks (**) indicate the bird was a wild-bred founder of the captive population.

| Sample | Year Sampled | Sex | Location |
| --- | --- | --- | --- |
| f724 | 1989 | F | Sutton |
| f725 | 1989 | M | Sutton |
| 041-48912† | 1995 | M | Armidale |
| 041-48942† | 1995 | M | Armidale |
| 041-48943 | 1995 | U | Armidale |
| 041-48951 | 1995 | F | Armidale |
| 041-48952† | 1995 | F | Armidale |
| 041-48953† | 1995 | M | Armidale |
| 041-87307† | 1995 | F | Canberra |
| 041-87308† | 1995 | M | Canberra |
| 041-87309† | 1995 | M | Canberra |
| 041-87310† | 1995 | M | Canberra |
| 041-87311† | 1995 | M | Canberra |
| 041-87312† | 1995 | F | Canberra |
| 041-87313† | 1995 | M | Canberra |
| 041-57161† | 1995 | M | Capertee |
| 041-57162† | 1995 | M | Capertee |
| 041-57163† | 1995 | M | Capertee |
| 041-57164† | 1995 | M | Capertee |
| 041-57165† | 1995 | M | Capertee |
| 041-57166† | 1995 | M | Capertee |
| 041-57167† | 1995 | M | Capertee |
| 041-57159† | 1995 | M | Capertee |
| 041-57168† | 1995 | F | Capertee |
| 041-57169 | 1995 | M | Capertee |
| 041-57170† | 1995 | M | Capertee |
| 041-57171† | 1995 | M | Capertee |
| 041-57172† | 1995 | M | Capertee |
| 041-57173† | 1995 | M | Capertee |
| 041-57176† | 1995 | F | Capertee |
| 041-57177† | 1995 | M | Capertee |
| 041-57178† | 1995 | F | Capertee |
| 041-57179† | 1995 | M | Capertee |
| 041-57180† | 1995 | M | Capertee |
| 041-57181 | 1995 | M | Capertee |
| 041-57183 | 1995 | U | Capertee |
| 041-87301† | 1995 | M | Chiltern |
| 041-87302† | 1995 | F | Chiltern |
| 041-87303† | 1995 | M | Chiltern |
| 041-87304† | 1995 | F | Chiltern |
| 041-87305† | 1995 | U | Chiltern |
| 041-87306† | 1995 | U | Chiltern |
| 041-48945† | 1996 | M | Armidale |
| 041-48946† | 1996 | M | Armidale |
| 041-48948† | 1996 | M | Armidale |
| 041-48983† | 1996 | F | Armidale |
| 041-48985† | 1996 | M | Armidale |
| 2-rhe yellow | 1996 | F | Capertee** |
| 4-rhe white | 1996 | F | Capertee** |
| 7-rhe orange | 1996 | M | Capertee** |
| 8-rhe yellow/orange | 1996 | M | Capertee** |
| 9-rhe black | 1996 | M | Capertee** |
| 1-rhe red | 1996 | M | Chiltern** |
| 3-rhe blue | 1996 | F | Chiltern** |
| 5-rhe purple | 1996 | F | Chiltern** |
| 6-rhe light green | 1996 | M | Chiltern** |
| 041-57204† | 1996 | F | Cumbo Rd, NSW |
| 041-57206† | 1996 | M | Cumbo Rd, NSW |
| 041-57207† | 1996 | F | Cumbo Rd, NSW |
| 041-57208† | 1996 | M | Goulburn River NP |
| 041-57209† | 1996 | M | Goulburn River NP |
| 041-57205† | 1996 | M | Munghorn East |
| 041-48986† | 1997 | F | Armidale |
| 041-48987† | 1997 | F | Armidale |
| 042-03901† | 1997 | M | Armidale |
| 042-03902† | 1997 | F | Armidale |
| 042-03907† | 1997 | F | Armidale |
| 042-03908† | 1997 | M | Armidale |
| 042-03909† | 1997 | M | Armidale |
| 042-03910† | 1997 | M | Armidale |
| 042-03911† | 1997 | M | Armidale |
| 042-03912† | 1997 | M | Armidale |
| 042-03914† | 1997 | M | Armidale |
| 042-03915† | 1997 | M | Armidale |
| 041-87401† | 1997 | U | Chiltern |
| 041-87402† | 1997 | M | Chiltern |
| 041-87403† | 1997 | M | Chiltern |
| 041-87404† | 1997 | U | Chiltern |
| 041-87405† | 1997 | M | Chiltern |
| 041-87406† | 1997 | M | Chiltern |
| 041-87407† | 1997 | M | Chiltern |
| 042-99716 | 2010 | F | Chiltern* |
| 042-99713 | 2010 | M | Chiltern* |
| 042-99755 | 2011 | F | Capertee |
| 042-99757 | 2011 | M | Capertee |
| 042-99703 | 2011 | M | Capertee |
| 042-99704 | 2011 | M | Capertee |
| 042-99753 | 2011 | F | Capertee |
| 042-99754 | 2011 | M | Indigo Valley |
| 042-99705 | 2011 | M | Lurg |
| 042-99767 | 2012 | M | Capertee |
| 042-99768 | 2012 | M | Capertee |
| 042-99769 | 2012 | M | Capertee |
| 042-99771 | 2012 | M | Capertee |
| 042-99773 | 2012 | M | Capertee |
| 042-99774 | 2012 | M | Capertee |
| 042-99775 | 2012 | M | Capertee |
| 042-99776 | 2012 | F | Capertee |
| 042-99777 | 2012 | M | Capertee |
| 042-99758 | 2012 | M | Quorrobolong |
| 042-99759 | 2012 | M | Quorrobolong |
| 042-99760 | 2012 | M | Quorrobolong |
| 042-99761 | 2012 | M | Quorrobolong |
| 042-99762 | 2012 | M | Quorrobolong |
| 042-99763 | 2012 | F | Quorrobolong |
| 042-99764 | 2012 | M | Quorrobolong |
| 042-99765 | 2012 | F | Quorrobolong |
| 042-99766 | 2012 | M | Quorrobolong |
| b00311 | 2012 | F | Taronga Zoo |
| b00433 | 2012 | F | Taronga Zoo |
| b00434 | 2012 | M | Taronga Zoo |
| b10402 | 2012 | M | Taronga Zoo |
| b10423 | 2012 | M | Taronga Zoo |
| b10428 | 2012 | F | Taronga Zoo |
| b10429 | 2012 | M | Taronga Zoo |
| b10490 | 2012 | M | Taronga Zoo |
| b20155 | 2012 | M | Taronga Zoo |
| b20156 | 2012 | F | Taronga Zoo |
| b20157 | 2012 | M | Taronga Zoo |
| b20158 | 2012 | M | Taronga Zoo |
| b20159 | 2012 | F | Taronga Zoo |
| b20211 | 2012 | M | Taronga Zoo |
| b20212 | 2012 | F | Taronga Zoo |
| b20213 | 2012 | F | Taronga Zoo |
| b20214 | 2012 | F | Taronga Zoo |
| b20215 | 2012 | F | Taronga Zoo |
| 990469 | 2012 | M | Taronga Zoo |
| a80336 | 2012 | M | Taronga Zoo |
| a80554 | 2012 | F | Taronga Zoo |
| a90185 | 2012 | M | Taronga Zoo |
| a80425 | 2012 | F | Taronga Zoo |
| a90391 | 2012 | F | Taronga Zoo |
| 970372 | 2012 | M | Taronga Zoo |
| a40334 | 2012 | M | Taronga Zoo |
| a60377 | 2012 | M | Taronga Zoo |
| a70780 | 2012 | F | Taronga Zoo |
| a80408 | 2012 | M | Taronga Zoo |
| a90282 | 2012 | F | Taronga Zoo |
| a60489 | 2012 | M | Taronga Zoo |
| a40333 | 2012 | F | Taronga Zoo |
| a90262 | 2012 | F | Taronga Zoo |
| a90284 | 2012 | M | Taronga Zoo |
| a90321 | 2012 | M | Taronga Zoo |
| a90372 | 2012 | M | Taronga Zoo |
| a90334 | 2013 | F | Taronga Zoo |
| a90406 | 2013 | F | Taronga Zoo |
| B10355 | 2013 | F | Taronga Zoo |
| b10370 | 2013 | F | Taronga Zoo |
| b10430 | 2013 | F | Taronga Zoo |
| B20237 | 2013 | M | Taronga Zoo |
| B20238 | 2013 | M | Taronga Zoo |
| B20239 | 2013 | M | Taronga Zoo |
| B20248 | 2013 | F | Taronga Zoo |
| B20252 | 2013 | M | Taronga Zoo |
| B20253 | 2013 | M | Taronga Zoo |
| B20259 | 2013 | M | Taronga Zoo |
| B20260 | 2013 | F | Taronga Zoo |
| B20261 | 2013 | F | Taronga Zoo |
| B20302 | 2013 | F | Taronga Zoo |
| B20303 | 2013 | F | Taronga Zoo |
| B20305 | 2013 | F | Taronga Zoo |
| B20331 | 2013 | M | Taronga Zoo |
| B20332 | 2013 | F | Taronga Zoo |
| B20333 | 2013 | M | Taronga Zoo |
| B20334 | 2013 | F | Taronga Zoo |
| B20335 | 2013 | M | Taronga Zoo |
| B20336 | 2013 | M | Taronga Zoo |
| B20337 | 2013 | F | Taronga Zoo |
| B20338 | 2013 | F | Taronga Zoo |
| B20348 | 2013 | M | Taronga Zoo |
| B20356 | 2013 | M | Taronga Zoo |
| B20357 | 2013 | F | Taronga Zoo |
| B20358 | 2013 | M | Taronga Zoo |
| B20389 | 2013 | F | Taronga Zoo |
| B20390 | 2013 | M | Taronga Zoo |
| B20392 | 2013 | M | Taronga Zoo |
| B20393 | 2013 | F | Taronga Zoo |
| B20399 | 2013 | M | Taronga Zoo |
| B20414 | 2013 | F | Taronga Zoo |
| B20415 | 2013 | M | Taronga Zoo |
| B20416 | 2013 | F | Taronga Zoo |
| B20417 | 2013 | F | Taronga Zoo |
| B20419 | 2013 | F | Taronga Zoo |
| B20420 | 2013 | M | Taronga Zoo |
| B20421 | 2013 | M | Taronga Zoo |
| B20427 | 2013 | F | Taronga Zoo |
| B20428 | 2013 | M | Taronga Zoo |
| B20435 | 2013 | M | Taronga Zoo |
| b20217 | no data | F | Taronga Zoo |
