# Supplementary material for: Very Low Population Structure in a Highly Mobile and Wide-Ranging Endangered Bird Species
Source: PLoS One. 2015 Dec 9;10(12):e0143746. doi: 10.1371/journal.pone.0143746 (PMC4674126; doi:10.1371/journal.pone.0143746)
Supplement: S2 Table — (DOCX) [file pone.0143746.s005.docx]

**S2 Table: List of microsatellite loci (with sources) and primers used in multiplex reactions.**

| **Locus** | **Multiplex**  **(Dye channel)** | **Primer Final (µM)** | **Design species** | **Reference** | **Forward Primer** | **Reverse Primer** | **Note** |
| --- | --- | --- | --- | --- | --- | --- | --- |
| BMC1 | H (800) | 0.10 | Bell Miner (*Manorina melanophrys*) | Painter et al. 1997 [83] | CTCATGGTGCTTATTTACGTGC | ACTAGCACATTTTGTCAGTTCC |  |
| BMC2 | H (700) | 0.04 | Bell Miner (*Manorina melanophrys*) | Painter et al. 1997 [83] | TCTGAGAGCCTTGGAAATGC | AGTTCACAGCAGAACTCCCG |  |
| BMC3 | H (800) | 0.20 | Bell Miner (*Manorina melanophrys*) | Painter et al. 1997 [83] | CCTGGCTGCCTGCACAGAC | TGAATTGCAGCTTCTGGGTGC |  |
| BMC4 | I2 (700) | 0.20 | Bell Miner (*Manorina melanophrys*) | Painter et al. 1997 [83] | GATAGGAGACTGAGAGACTGTCCC | TTTCTGAAGGGTTAGCTACAGACC | Unable to be scored reliably |
| FhU2 | H (800) | 0.02 | European Pied Flycatcher (*Ficedula hypoleuca*) | Primmer et al. 1996 [84] | GTGTTCTTAAAACATGCCTGGAGG | GCACAGGTAAATATTTGCTGGGCC |  |
| HrU2 | I2 (800) | 0.20 | Barn swallow (*Hirundo rustica*) | Primmer et al. 1995 [85] | CATCAAGAGAGGGATGGAAAGAGG | GAAAAGATTATTTTTCTTTCTCCC |  |
| McYm7 | I2 (800) | 0.02 | Superb fairy-wren (*Malurus cyaneus*) | Double et al. 1997 [86] | CTTTGTGTTGCTGTTAGGTAGAA | GGCTCAACAGCTATTTGCAT |  |
| Pn1 | M2 (800) | 0.02 | New Holland honeyeater (*Phylidonyris novaehollandiae*) | Myers et al. 2009 [87] | CCACATCGTAGAAGGAAAGACC | CACCATACCTCCTTTGCATTCCTCC |  |
| Pn12 | N2 (800) | 0.01 | New Holland honeyeater (*Phylidonyris novaehollandiae*) | Myers et al. 2009 [87] | ACTGCTTGAGGAGGGATGTG | TGCCAGTCCGTTGGGAAATAC |  |
| Pn13 | N2 (800) | 0.20 | New Holland honeyeater (*Phylidonyris novaehollandiae*) | Myers et al. 2009 [87] | AAAGAGGGAGTGGTGGTATG | AAACGGCCAGTCAGATGTGTAG |  |
| Pn15 | N2 (700) | 0.10 | New Holland honeyeater (*Phylidonyris novaehollandiae*) | Myers et al. 2009 [87] | AGAAGAGCCTCCAGACCACA | TTGGGAAAGTCTCAACTGGC |  |
| Pn2 | M2 (800) | 0.10 | New Holland honeyeater (*Phylidonyris novaehollandiae*) | Myers et al. 2009 [87] | GGCTCTTGAGAGGACAAGAAA | CTCATCCCTCTTCTCTGGAATG |  |
| Pn23 | N2 (800) | 0.01 | New Holland honeyeater (*Phylidonyris novaehollandiae*) | Myers et al. 2009 [87] | AAAGTCTGACACTGCCTCTCC | TGGGGAACTGAACTCATCCT |  |
| Pn3 | M2 (800) | 0.01 | New Holland honeyeater (*Phylidonyris novaehollandiae*) | Myers et al. 2009 [87] | AGTTTTTGTGGTGGGAGCAG | GGTGCAAACTCAGACACAGAAG |  |
| Pn5 | M2 (700) | 0.10 | New Holland honeyeater (*Phylidonyris novaehollandiae*) | Myers et al. 2009 [87] | CTGTCCTTTCATCACTTTCATC | CAGGTTTGTTTTCAGCAGCA |  |
| Pocco8 | H (700) | 0.16 | Crowned leaf warbler (*Phylloscopus occipitalis*) | Bensch et al. 1997 [88] | GCATGTCTCTTCAGACATCTGC | ATGTAGAGCTCCCATGGTGG |  |
| P2/P8 (Sexing) | - | 0.125 | Chicken (*Gallus gallus domesticus*) | Griffiths et al. 1998 [89] | CTCCCAAGGATGAGRAAYTG | TCTGCATCGCTAAATCCTTT |  |

**References:**

[83] Painter J, Crozier RH, Crozier YC, Clarke MF. Characterization of microsatellite loci for a co-operatively breeding honeyeater. Molecular Ecology 1997; 6:1103-1105.

[84] Primmer CR, Møller AP, Ellegren H. A wide-range survey of cross-species microsatellite amplification in birds. Molecular Ecology 1996; 5:365-378.

[85] Primmer, CR, Møller AP, Ellegren H. Resolving genetic relationships with microsatellite markers: A parentage testing system for the swallow *Hirundo rustica*. Molecular Ecology 1995; 4:493-498.

[86] Double MC, Dawson D, Burke T, Cockburn A. Finding the fathers in the least faithful bird: A microsatellite-based genotyping system for the superb fairy-wren *Malurus cyaneus*. Molecular Ecology 1997; 6:691-693.

[87] Myers SA, Gardner MG, Donnellan S, Kleindorfer S. New Holland honeyeater (*Phylidonyris novaehollandiae*) microsatellites: Isolation and characterization of 15 novel markers using an enrichment method. Molecular Ecology Resources 2009; 9:2052-1054.

[88] Bensch S, Price T, Kohn J. Isolation and characterization of microsatellite loci in a *Phylloscopus* warbler. Molecular Ecology 1997; 6:91-92.

[89] Griffiths R, Double MC, Orr K, Dawson JG. A DNA test to sex most birds. Molecular Ecology 1998; 7:1071-1075.
