# Supplementary material for: Very Low Population Structure in a Highly Mobile and Wide-Ranging Endangered Bird Species
Source: PLoS One. 2015 Dec 9;10(12):e0143746. doi: 10.1371/journal.pone.0143746 (PMC4674126; doi:10.1371/journal.pone.0143746)
Supplement: S3 Table — Table a includes all wild and captive birds (N = 189) in calculations for the number and size of alleles; Hardy-Weinberg and F IS values are calculated using only wild birds (N = 108) treated as one population. Table b shows the probability of significance of Hardy-Weinberg tests (only significant results shown) and F IS values when birds are treated as six populations based on where they were captured. Locus HrU2 did not have enough allelic diversity to calculate F IS at Canberra (monomorphic except for one allele in 1 individual). (DOCX) [file pone.0143746.s006.docx]

**S3 Table: List of 15 loci used in analyses and the number of alleles, allele size, probability of significance for Hardy-Weinberg tests (only significant results shown), and *F*_IS_ values.** Table a includes all wild and captive birds (N=189) in calculations for the number and size of alleles; Hardy-Weinberg and *F*_IS_ values are calculated using only wild birds (N=108) treated as one population. Table b shows the probability of significance of Hardy-Weinberg tests (only significant results shown) and *F*_IS_ values when birds are treated as six populations based on where they were captured. Locus HrU2 did not have enough allelic diversity to calculate *F*_IS_ at Canberra (monomorphic except for one allele in 1 individual).

a)

| **Locus** | **No of Alleles** | **Product Range (BP)** | **P (HW)** | ***F*_IS_** |
| --- | --- | --- | --- | --- |
| **BMC1** | 19 | 187-227 |  | 0.0220 |
| **BMC2** | 4 | 179-185 | p<0.001 | 0.1998 |
| **BMC3** | 1 | 96 |  | monomorphic |
| **FhU2** | 1 | 160 |  | monomorphic |
| **Pocco8** | 10 | 226-274 |  | 0.0239 |
| **Pn1** | 14 | 296-328 | p<0.001 | 0.2133 |
| **Pn2** | 1 | 108 |  | monomorphic |
| **Pn3** | 3 | 234-238 |  | -0.0133 |
| **Pn5** | 3 | 258-262 |  | 0.1022 |
| **Pn12** | 1 | 252 |  | monomorphic |
| **Pn13** | 7 | 324-338 |  | 0.1244 |
| **Pn15** | 3 | 198-294 |  | -0.0653 |
| **Pn23** | 8 | 157-171 |  | 0.0200 |
| **HrU2** | 5 | 146-154 |  | 0.1000 |
| **McYm7** | 1 | 106 |  | monomorphic |

b)

|  | **Armidale (N=23)** | | **Canberra (N=9)** | | **Capertee (N=40)** | | **Chiltern (N=21)** | | **Goulburn River (N=6)** | | **Quorrobolong (N=9)** | |
| --- | --- | --- | --- | --- | --- | --- | --- | --- | --- | --- | --- | --- |
|  | **P(HW)** | ***F*_IS_** | **P(HW)** | ***F*_IS_** | **P(HW)** | ***F*_IS_** | **P(HW)** | ***F*_IS_** | **P(HW)** | ***F*_IS_** | **P(HW)** | ***F*_IS_** |
| **BMC1** |  | -0.0096 |  | -0.0566 |  | 0.0836 |  | -0.0662 |  | 0.0566 |  | 0 |
| **BMC2** | p<0.05 | 0.2816 |  | -0.1636 |  | 0.0989 | p<0.01 | 0.6104 |  | monomorphic |  | -0.1429 |
| **BMC3** |  | monomorphic |  | monomorphic |  | monomorphic |  | monomorphic |  | monomorphic |  | Monomorphic |
| **FhU2** |  | monomorphic |  | monomorphic |  | monomorphic |  | monomorphic |  | monomorphic |  | Monomorphic |
| **Pocco8** |  | 0.0935 |  | 0.5897 |  | -0.0154 |  | -0.154 |  | 0.1304 |  | -0.0526 |
| **Pn1** | p<0.001 | 0.1086 |  | 0.1765 | p<0.05 | 0.3337 |  | 0.1176 |  | 0.1579 |  | 0.0968 |
| **Pn2** |  | monomorphic |  | monomorphic |  | monomorphic |  | monomorphic |  | monomorphic |  | Monomorphic |
| **Pn3** |  | -0.0746 |  | -0.0667 |  | 0.0886 |  | -0.0625 |  | monomorphic |  | -0.0909 |
| **Pn5** |  | 0.3293 |  | 0.2258 |  | -0.0879 |  | 0.1682 |  | 0.0625 |  | -0.1111 |
| **Pn12** |  | monomorphic |  | monomorphic |  | monomorphic |  | monomorphic |  | monomorphic |  | monomorphic |
| **Pn13** |  | 0.0023 |  | -0.0667 |  | 0.1807 |  | 0.0476 |  | -0.0588 |  | 0.5152 |
| **Pn15** |  | -0.1892 |  | monomorphic |  | 0.0161 |  | -0.1111 |  | -0.25 |  | monomorphic |
| **Pn23** |  | -0.1221 |  | 0.1765 |  | 0.1041 |  | 0.0233 |  | monomorphic |  | 0.068 |
| **HrU2** |  | -0.0256 |  | no value |  | -0.0507 | p<0.05 | 0.6512 |  | monomorphic |  | -0.0667 |
| **McYm7** |  | monomorphic |  | monomorphic |  | monomorphic |  | monomorphic |  | monomorphic |  | monomorphic |
