# Supplementary material for: Very Low Population Structure in a Highly Mobile and Wide-Ranging Endangered Bird Species
Source: PLoS One. 2015 Dec 9;10(12):e0143746. doi: 10.1371/journal.pone.0143746 (PMC4674126; doi:10.1371/journal.pone.0143746)
Supplement: S4 Table — (DOCX) [file pone.0143746.s007.docx]

**S4 Table: Allele frequencies for each locus by site.**

| **Locus** | **Allele/n** | **Armidale** | **Canberra** | **Capertee** | **Chiltern** | **Goulburn River** | **Quorrobolong** | **Taronga Zoo** |
| --- | --- | --- | --- | --- | --- | --- | --- | --- |
| **BMC1A** | **N** | 22 | 8 | 38 | 14 | 6 | 9 | 92 |
|  | **187** | 0.000 | 0.000 | 0.013 | 0.000 | 0.000 | 0.000 | 0.011 |
|  | **189** | 0.159 | 0.188 | 0.171 | 0.214 | 0.250 | 0.278 | 0.152 |
|  | **193** | 0.000 | 0.000 | 0.013 | 0.000 | 0.000 | 0.000 | 0.005 |
|  | **195** | 0.136 | 0.063 | 0.039 | 0.036 | 0.250 | 0.056 | 0.103 |
|  | **197** | 0.136 | 0.063 | 0.039 | 0.000 | 0.000 | 0.056 | 0.011 |
|  | **199** | 0.091 | 0.063 | 0.105 | 0.179 | 0.167 | 0.056 | 0.071 |
|  | **201** | 0.000 | 0.188 | 0.013 | 0.000 | 0.000 | 0.222 | 0.000 |
|  | **203** | 0.023 | 0.063 | 0.013 | 0.000 | 0.000 | 0.000 | 0.005 |
|  | **205** | 0.000 | 0.000 | 0.026 | 0.000 | 0.000 | 0.000 | 0.000 |
|  | **207** | 0.045 | 0.063 | 0.132 | 0.071 | 0.083 | 0.056 | 0.098 |
|  | **209** | 0.091 | 0.063 | 0.105 | 0.143 | 0.167 | 0.056 | 0.212 |
|  | **211** | 0.023 | 0.063 | 0.105 | 0.071 | 0.000 | 0.000 | 0.060 |
|  | **213** | 0.182 | 0.063 | 0.066 | 0.214 | 0.000 | 0.111 | 0.190 |
|  | **215** | 0.045 | 0.000 | 0.053 | 0.036 | 0.083 | 0.056 | 0.005 |
|  | **217** | 0.045 | 0.000 | 0.039 | 0.000 | 0.000 | 0.056 | 0.065 |
|  | **219** | 0.023 | 0.063 | 0.013 | 0.036 | 0.000 | 0.000 | 0.005 |
|  | **223** | 0.000 | 0.000 | 0.013 | 0.000 | 0.000 | 0.000 | 0.005 |
|  | **225** | 0.000 | 0.063 | 0.026 | 0.000 | 0.000 | 0.000 | 0.000 |
|  | **227** | 0.000 | 0.000 | 0.013 | 0.000 | 0.000 | 0.000 | 0.000 |
| **BMC2A** | **N** | 23 | 9 | 40 | 21 | 6 | 9 | 92 |
|  | **179** | 0.065 | 0.167 | 0.163 | 0.167 | 0.000 | 0.000 | 0.196 |
|  | **181** | 0.870 | 0.778 | 0.725 | 0.786 | 1.000 | 0.833 | 0.592 |
|  | **183** | 0.000 | 0.000 | 0.013 | 0.000 | 0.000 | 0.000 | 0.005 |
|  | **185** | 0.065 | 0.056 | 0.100 | 0.048 | 0.000 | 0.167 | 0.207 |
| **BMC3A** | **N** | 22 | 8 | 38 | 15 | 6 | 9 | 92 |
|  | **96** | 1.000 | 1.000 | 1.000 | 1.000 | 1.000 | 1.000 | 1.000 |
| **FhU2A** | **N** | 22 | 8 | 38 | 15 | 6 | 9 | 92 |
|  | **160** | 1.000 | 1.000 | 1.000 | 1.000 | 1.000 | 1.000 | 1.000 |
| **Pocco8A** | **N** | 23 | 9 | 39 | 20 | 6 | 9 | 91 |
|  | **226** | 0.022 | 0.000 | 0.013 | 0.000 | 0.000 | 0.000 | 0.000 |
|  | **230** | 0.500 | 0.556 | 0.487 | 0.525 | 0.500 | 0.667 | 0.604 |
|  | **232** | 0.370 | 0.444 | 0.321 | 0.350 | 0.167 | 0.222 | 0.159 |
|  | **234** | 0.000 | 0.000 | 0.000 | 0.000 | 0.083 | 0.000 | 0.000 |
|  | **242** | 0.043 | 0.000 | 0.090 | 0.075 | 0.083 | 0.056 | 0.077 |
|  | **246** | 0.000 | 0.000 | 0.013 | 0.000 | 0.083 | 0.000 | 0.000 |
|  | **262** | 0.000 | 0.000 | 0.026 | 0.000 | 0.000 | 0.000 | 0.000 |
|  | **268** | 0.022 | 0.000 | 0.000 | 0.000 | 0.083 | 0.000 | 0.000 |
|  | **272** | 0.043 | 0.000 | 0.051 | 0.050 | 0.000 | 0.000 | 0.159 |
|  | **274** | 0.000 | 0.000 | 0.000 | 0.000 | 0.000 | 0.056 | 0.000 |
| **Pn1A** | **N** | 21 | 8 | 33 | 13 | 5 | 9 | 85 |
|  | **296** | 0.024 | 0.000 | 0.030 | 0.000 | 0.000 | 0.000 | 0.000 |
|  | **304** | 0.024 | 0.000 | 0.000 | 0.000 | 0.000 | 0.000 | 0.000 |
|  | **306** | 0.024 | 0.000 | 0.000 | 0.000 | 0.100 | 0.000 | 0.018 |
|  | **308** | 0.143 | 0.188 | 0.136 | 0.038 | 0.200 | 0.000 | 0.012 |
|  | **310** | 0.143 | 0.250 | 0.167 | 0.192 | 0.200 | 0.167 | 0.100 |
|  | **312** | 0.000 | 0.063 | 0.227 | 0.231 | 0.000 | 0.222 | 0.253 |
|  | **314** | 0.143 | 0.125 | 0.030 | 0.000 | 0.000 | 0.000 | 0.018 |
|  | **316** | 0.286 | 0.063 | 0.030 | 0.038 | 0.200 | 0.056 | 0.059 |
|  | **318** | 0.000 | 0.063 | 0.106 | 0.154 | 0.000 | 0.278 | 0.141 |
|  | **320** | 0.048 | 0.125 | 0.015 | 0.077 | 0.100 | 0.056 | 0.018 |
|  | **322** | 0.143 | 0.125 | 0.197 | 0.192 | 0.000 | 0.167 | 0.329 |
|  | **324** | 0.000 | 0.000 | 0.045 | 0.077 | 0.100 | 0.056 | 0.047 |
|  | **326** | 0.024 | 0.000 | 0.000 | 0.000 | 0.100 | 0.000 | 0.000 |
|  | **328** | 0.000 | 0.000 | 0.015 | 0.000 | 0.000 | 0.000 | 0.006 |
| **Pn2A** | **N** | 22 | 9 | 40 | 21 | 6 | 9 | 92 |
|  | **108** | 1.000 | 1.000 | 1.000 | 1.000 | 1.000 | 1.000 | 1.000 |
| **Pn3A** | **N** | 21 | 9 | 37 | 18 | 6 | 9 | 90 |
|  | **234** | 0.762 | 0.889 | 0.811 | 0.778 | 0.917 | 0.833 | 0.872 |
|  | **236** | 0.095 | 0.111 | 0.135 | 0.194 | 0.000 | 0.111 | 0.122 |
|  | **238** | 0.143 | 0.000 | 0.054 | 0.028 | 0.083 | 0.056 | 0.006 |
| **Pn5A** | **N** | 23 | 9 | 39 | 18 | 6 | 9 | 92 |
|  | **258** | 0.804 | 0.722 | 0.795 | 0.556 | 0.583 | 0.611 | 0.614 |
|  | **260** | 0.196 | 0.278 | 0.205 | 0.417 | 0.417 | 0.389 | 0.386 |
|  | **262** | 0.000 | 0.000 | 0.000 | 0.028 | 0.000 | 0.000 | 0.000 |
| **Pn12A** | **N** | 20 | 9 | 38 | 15 | 6 | 9 | 88 |
|  | **252** | 1.000 | 1.000 | 1.000 | 1.000 | 1.000 | 1.000 | 1.000 |
| **Pn13A** | **N** | 19 | 9 | 35 | 11 | 4 | 9 | 83 |
|  | **324** | 0.000 | 0.056 | 0.014 | 0.000 | 0.000 | 0.000 | 0.000 |
|  | **326** | 0.053 | 0.111 | 0.086 | 0.227 | 0.000 | 0.056 | 0.030 |
|  | **328** | 0.526 | 0.611 | 0.471 | 0.545 | 0.500 | 0.556 | 0.602 |
|  | **330** | 0.316 | 0.056 | 0.257 | 0.091 | 0.250 | 0.167 | 0.139 |
|  | **332** | 0.053 | 0.111 | 0.043 | 0.045 | 0.250 | 0.000 | 0.066 |
|  | **334** | 0.026 | 0.056 | 0.129 | 0.045 | 0.000 | 0.167 | 0.163 |
|  | **338** | 0.026 | 0.000 | 0.000 | 0.045 | 0.000 | 0.056 | 0.000 |
| **Pn15A** | **N** | 23 | 9 | 40 | 21 | 6 | 9 | 92 |
|  | **198** | 0.174 | 0.000 | 0.150 | 0.119 | 0.250 | 0.000 | 0.152 |
|  | **200** | 0.000 | 0.000 | 0.013 | 0.000 | 0.000 | 0.000 | 0.000 |
|  | **204** | 0.826 | 1.000 | 0.838 | 0.881 | 0.750 | 1.000 | 0.848 |
| **Pn23A** | **N** | 19 | 9 | 38 | 15 | 6 | 9 | 90 |
|  | **157** | 0.000 | 0.000 | 0.013 | 0.000 | 0.000 | 0.000 | 0.000 |
|  | **159** | 0.079 | 0.000 | 0.000 | 0.033 | 0.000 | 0.000 | 0.006 |
|  | **161** | 0.105 | 0.111 | 0.105 | 0.100 | 0.000 | 0.222 | 0.206 |
|  | **163** | 0.632 | 0.722 | 0.605 | 0.600 | 0.917 | 0.500 | 0.450 |
|  | **165** | 0.184 | 0.111 | 0.197 | 0.133 | 0.083 | 0.111 | 0.278 |
|  | **167** | 0.000 | 0.056 | 0.066 | 0.133 | 0.000 | 0.111 | 0.061 |
|  | **169** | 0.000 | 0.000 | 0.000 | 0.000 | 0.000 | 0.056 | 0.000 |
|  | **171** | 0.000 | 0.000 | 0.013 | 0.000 | 0.000 | 0.000 | 0.000 |
| **HrU2A** | **N** | 21 | 9 | 39 | 16 | 6 | 9 | 92 |
|  | **146** | 0.000 | 0.056 | 0.051 | 0.094 | 0.000 | 0.000 | 0.033 |
|  | **148** | 0.000 | 0.000 | 0.000 | 0.000 | 0.000 | 0.000 | 0.005 |
|  | **150** | 0.952 | 0.944 | 0.923 | 0.906 | 1.000 | 0.889 | 0.886 |
|  | **152** | 0.048 | 0.000 | 0.026 | 0.000 | 0.000 | 0.111 | 0.071 |
|  | **154** | 0.000 | 0.000 | 0.000 | 0.000 | 0.000 | 0.000 | 0.005 |
| **McYm7A** | **N** | 21 | 9 | 38 | 16 | 6 | 9 | 91 |
|  | **106** | 1.000 | 1.000 | 1.000 | 1.000 | 1.000 | 1.000 | 1.000 |
