# Supplementary material for: Very Low Population Structure in a Highly Mobile and Wide-Ranging Endangered Bird Species
Source: PLoS One. 2015 Dec 9;10(12):e0143746. doi: 10.1371/journal.pone.0143746 (PMC4674126; doi:10.1371/journal.pone.0143746)
Supplement: S5 Table — Values in parentheses are standard errors. (DOCX) [file pone.0143746.s008.docx]

**S5 Table: Allelic richness (AR) by geographic location for each polymorphic locus.** Values in parentheses are standard errors.

| **Locus** | **Armidale** | **Canberra** | **Capertee** | **Chiltern** | **Goulburn River** | **Quorrobolong** |
| --- | --- | --- | --- | --- | --- | --- |
| **N** | **23** | **9** | **40** | **21** | **6** | **9** |
| **BMC1** | 5.739 | 6.800 | 6.115 | 5.252 | 5.115 | 5.719 |
| **BMC2** | 1.889 | 2.297 | 2.462 | 2.149 | 1.000 | 1.853 |
| **Pocco8** | 2.971 | 1.999 | 3.263 | 2.841 | 4.576 | 2.820 |
| **Pn1** | 5.035 | 5.662 | 5.105 | 5.128 | 6.133 | 4.941 |
| **Pn3** | 2.329 | 1.706 | 2.080 | 2.080 | 1.667 | 2.150 |
| **Pn5** | 1.852 | 1.971 | 1.856 | 2.215 | 2.000 | 1.996 |
| **Pn13** | 3.151 | 3.745 | 3.559 | 3.621 | 3.000 | 3.595 |
| **Pn15** | 1.813 | 1.000 | 1.845 | 1.673 | 1.982 | 1.000 |
| **Pn23** | 2.986 | 2.856 | 3.098 | 3.353 | 1.667 | 3.787 |
| **HrU2** | 1.348 | 1.444 | 1.553 | 1.592 | 1.000 | 1.706 |
| **Mean AR** | 2.911 (0.456) | 2.948 (0.603) | 3.094 (0.475) | 2.990 (0.422) | 2.814  (0.578) | 2.957  (0.482) |
