# Supplementary material for: Very Low Population Structure in a Highly Mobile and Wide-Ranging Endangered Bird Species
Source: PLoS One. 2015 Dec 9;10(12):e0143746. doi: 10.1371/journal.pone.0143746 (PMC4674126; doi:10.1371/journal.pone.0143746)
Supplement: S6 Table — Values in parentheses are standard errors. (DOCX) [file pone.0143746.s009.docx]

**S6 Table: Allelic richness (AR) and observed (H_O_) and expected (H_E_) heterozygosities for polymorphic loci in the wild and captive populations.** Values in parentheses are standard errors.

| **Population** | **Locus** | **N** | **AR** | **H_O_** | **H_E_** |
| --- | --- | --- | --- | --- | --- |
| **Wild** | **BMC1** | 97 | 18.397 | 0.887 | 0.902 |
|  | **BMC2** | 108 | 3.769 | 0.278 | 0.345 |
|  | **Pocco8** | 106 | 9.381 | 0.604 | 0.616 |
|  | **Pn1** | 89 | 13.857 | 0.697 | 0.879 |
|  | **Pn3** | 100 | 3.000 | 0.330 | 0.324 |
|  | **Pn5** | 104 | 2.798 | 0.365 | 0.405 |
|  | **Pn13** | 87 | 6.998 | 0.586 | 0.665 |
|  | **Pn15** | 108 | 2.769 | 0.250 | 0.234 |
|  | **Pn23** | 96 | 7.593 | 0.552 | 0.560 |
|  | **HrU2** | 100 | 3.000 | 0.120 | 0.133 |
|  | **Pop Mean** | 99.5 | 7.156 (1.702) | 0.467 (0.075) | 0.506 (0.083) |
| **Captive** | **BMC1** | 92 | 14.493 | 0.913 | 0.862 |
|  | **BMC2** | 92 | 3.902 | 0.565 | 0.568 |
|  | **Pocco8** | 91 | 4.000 | 0.571 | 0.578 |
|  | **Pn1** | 85 | 10.976 | 0.576 | 0.791 |
|  | **Pn3** | 90 | 2.922 | 0.256 | 0.224 |
|  | **Pn5** | 92 | 2.000 | 0.424 | 0.474 |
|  | **Pn13** | 83 | 5.000 | 0.386 | 0.586 |
|  | **Pn15** | 92 | 2.000 | 0.261 | 0.258 |
|  | **Pn23** | 90 | 4.922 | 0.711 | 0.674 |
|  | **HrU2** | 92 | 4.804 | 0.196 | 0.209 |
|  | **Pop Mean** | 89.9 | 5.502 (1.282) | 0.486 (0.071) | 0.523 (0.073) |
