# Supplementary material for: Very Low Population Structure in a Highly Mobile and Wide-Ranging Endangered Bird Species
Source: PLoS One. 2015 Dec 9;10(12):e0143746. doi: 10.1371/journal.pone.0143746 (PMC4674126; doi:10.1371/journal.pone.0143746)
Supplement: S7 Table — N is the number of sampled individuals in the time period, HO is the observed heterozygosity, HE is the expected heterozygosity, and No private alleles refers to the number of alleles found only in samples from one time period. Note this analysis includes only polymorphic loci. Standard errors are given in parentheses. (DOCX) [file pone.0143746.s010.docx]

**S7 Table: Allelic richness (AR) and heterozygosity for wild regent honeyeaters sampled before 2000 and after 2010.** N is the number of sampled individuals in the time period, H_O_ is the observed heterozygosity, H_E_ is the expected heterozygosity, and No private alleles refers to the number of alleles found only in samples from one time period. Note this analysis includes only polymorphic loci. Standard errors are given in parentheses.

| **Sample** | **Pre-2000** | **Post-2010** |
| --- | --- | --- |
| **N** | 81 | 27 |
| **BMC1** | 14.500 | 14.000 |
| **BMC2** | 3.324 | 3.000 |
| **Pocco8** | 5.738 | 8.000 |
| **Pn1** | 11.126 | 9.000 |
| **Pn3** | 2.986 | 3.000 |
| **Pn5** | 2.351 | 2.000 |
| **Pn13** | 6.141 | 7.000 |
| **Pn15** | 2.000 | 3.000 |
| **Pn23** | 5.210 | 6.000 |
| **HrU2** | 2.717 | 3.000 |
| **Mean AR** | 5.609 (1.308) | 5.800 (1.200) |
| **Mean H_O_** | 0.462 (0.076) | 0.485 (0.075) |
| **Mean H_E_** | 0.499 (0.083) | 0.505 (0.079) |
| **No Private Alleles** | 15 | 6 |
