# Supplementary material for: Very Low Population Structure in a Highly Mobile and Wide-Ranging Endangered Bird Species
Source: PLoS One. 2015 Dec 9;10(12):e0143746. doi: 10.1371/journal.pone.0143746 (PMC4674126; doi:10.1371/journal.pone.0143746)
Supplement: S8 Table — Alleles with a frequency of zero in one of the samples are shaded gray; such alleles may represent a sampling issue or, if not found in the post-2010 sample, extinction. (DOCX) [file pone.0143746.s011.docx]

**S8 Table: List of allele frequencies for each polymorphic locus for wild-bred birds sampled pre-2000 and post-2010.** Alleles with a frequency of zero in one of the samples are shaded gray; such alleles may represent a sampling issue or, if not found in the post-2010 sample, extinction.

| **Locus** | **Allele/n** | **Pre-2000** | **Post-2010** |
| --- | --- | --- | --- |
| **BMC1** | **N** | 70 | 25 |
|  | **187** | 0.007 | 0.000 |
|  | **189** | 0.193 | 0.180 |
|  | **193** | 0.007 | 0.000 |
|  | **195** | 0.079 | 0.080 |
|  | **197** | 0.057 | 0.060 |
|  | **199** | 0.100 | 0.140 |
|  | **201** | 0.021 | 0.100 |
|  | **203** | 0.014 | 0.020 |
|  | **205** | 0.014 | 0.000 |
|  | **207** | 0.086 | 0.100 |
|  | **209** | 0.093 | 0.120 |
|  | **211** | 0.079 | 0.020 |
|  | **213** | 0.121 | 0.060 |
|  | **215** | 0.043 | 0.060 |
|  | **217** | 0.036 | 0.020 |
|  | **219** | 0.029 | 0.000 |
|  | **223** | 0.007 | 0.000 |
|  | **225** | 0.014 | 0.020 |
|  | **227** | 0.000 | 0.020 |
| **BMC2** | **N** | 81 | 25 |
|  | **179** | 0.148 | 0.040 |
|  | **181** | 0.778 | 0.840 |
|  | **183** | 0.006 | 0.000 |
|  | **185** | 0.068 | 0.120 |
| **Pocco8** | **N** | 79 | 25 |
|  | **226** | 0.006 | 0.020 |
|  | **230** | 0.525 | 0.520 |
|  | **232** | 0.348 | 0.280 |
|  | **234** | 0.006 | 0.000 |
|  | **242** | 0.063 | 0.080 |
|  | **246** | 0.006 | 0.020 |
|  | **262** | 0.006 | 0.020 |
|  | **268** | 0.013 | 0.000 |
|  | **272** | 0.025 | 0.040 |
|  | **274** | 0.000 | 0.020 |
| **Pn1** | **N** | 62 | 25 |
|  | **296** | 0.016 | 0.020 |
|  | **304** | 0.008 | 0.000 |
|  | **306** | 0.016 | 0.000 |
|  | **308** | 0.169 | 0.000 |
|  | **310** | 0.218 | 0.080 |
|  | **312** | 0.065 | 0.360 |
|  | **314** | 0.081 | 0.000 |
|  | **316** | 0.137 | 0.020 |
|  | **318** | 0.056 | 0.180 |
|  | **320** | 0.056 | 0.040 |
|  | **322** | 0.145 | 0.180 |
|  | **324** | 0.016 | 0.100 |
|  | **326** | 0.016 | 0.000 |
|  | **328** | 0.000 | 0.020 |
| **Pn3** | **N** | 73 | 25 |
|  | **234** | 0.836 | 0.740 |
|  | **236** | 0.103 | 0.180 |
|  | **238** | 0.062 | 0.080 |
| **Pn5** | **N** | 77 | 25 |
|  | **258** | 0.734 | 0.700 |
|  | **260** | 0.260 | 0.300 |
|  | **262** | 0.006 | 0.000 |
| **Pn13** | **N** | 60 | 25 |
|  | **324** | 0.008 | 0.020 |
|  | **326** | 0.075 | 0.140 |
|  | **328** | 0.483 | 0.560 |
|  | **330** | 0.258 | 0.140 |
|  | **332** | 0.075 | 0.020 |
|  | **334** | 0.083 | 0.100 |
|  | **338** | 0.017 | 0.020 |
| **Pn15** | **N** | 81 | 25 |
|  | **198** | 0.136 | 0.100 |
|  | **200** | 0.000 | 0.020 |
|  | **204** | 0.864 | 0.880 |
| **Pn23** | **N** | 69 | 25 |
|  | **157** | 0.007 | 0.000 |
|  | **159** | 0.029 | 0.000 |
|  | **161** | 0.094 | 0.140 |
|  | **163** | 0.652 | 0.580 |
|  | **165** | 0.174 | 0.120 |
|  | **167** | 0.043 | 0.120 |
|  | **169** | 0.000 | 0.020 |
|  | **171** | 0.000 | 0.020 |
| **HrU2** | **N** | 73 | 25 |
|  | **146** | 0.048 | 0.000 |
|  | **150** | 0.932 | 0.940 |
|  | **152** | 0.021 | 0.060 |
