# Supplementary material for: Very Low Population Structure in a Highly Mobile and Wide-Ranging Endangered Bird Species
Source: PLoS One. 2015 Dec 9;10(12):e0143746. doi: 10.1371/journal.pone.0143746 (PMC4674126; doi:10.1371/journal.pone.0143746)
Supplement: S9 Table — An excess indicates a recent population size bottleneck. (DOCX) [file pone.0143746.s012.docx]

**S9 Table: Estimated heterozygosity excess or deficit as calculated by the two-phase model in BOTTLENECK for regent honeyeaters captured in the wild for a) all loci** **together using the Wilcoxon test and b) measured heterozygosity (H_e_) and heterozygosity at mutation-drift equilibrium (H_eq_) for each polymorphic locus.** An excess indicates a recent population size bottleneck.

a)

| **Pre-2000 Samples** |  |
| --- | --- |
| Probability (one tail for heterozygosity deficiency) | 0.161 |
| Probability (one tail for heterozygosity excess) | 0.862 |
| Probability (two tails for heterozygosity excess and deficiency) | 0.322 |
| **Post-2010 Samples** |  |
| Probability (one tail for heterozygosity deficiency) | 0.097 |
| Probability (one tail for heterozygosity excess) | 0.920 |
| Probability (two tails for heterozygosity excess and deficiency) | 0.193 |

b)

| **Population** | **Locus** | **H_e_** | **H_eq_** |
| --- | --- | --- | --- |
| Pre-2000 | BMC1 | 0.908 | 0.891 |
|  | BMC2 | 0.371 | 0.480 |
|  | Pocco8 | 0.602 | 0.757 |
|  | Pn1 | 0.873 | 0.844 |
|  | Pn3 | 0.289 | 0.374 |
|  | Pn5 | 0.397 | 0.363 |
|  | Pn13 | 0.687 | 0.690 |
|  | Pn15 | 0.236 | 0.194 |
|  | Pn23 | 0.537 | 0.636 |
|  | HrU2 | 0.130 | 0.371 |
| Post-2010 | BMC1 | 0.910 | 0.887 |
|  | BMC2 | 0.266 | 0.423 |
|  | Pocco8 | 0.673 | 0.777 |
|  | Pn1 | 0.811 | 0.801 |
|  | Pn3 | 0.419 | 0.415 |
|  | Pn5 | 0.440 | 0.229 |
|  | Pn13 | 0.617 | 0.734 |
|  | Pn15 | 0.234 | 0.419 |
|  | Pn23 | 0.630 | 0.693 |
|  | HrU2 | 0.142 | 0.426 |
