# Supplementary material for: Very Low Population Structure in a Highly Mobile and Wide-Ranging Endangered Bird Species
Source: PLoS One. 2015 Dec 9;10(12):e0143746. doi: 10.1371/journal.pone.0143746 (PMC4674126; doi:10.1371/journal.pone.0143746)
Supplement: S10 Table — The mean, range, and standard deviation (SD) for F ST represent that of 10 runs, and the number of those runs with significant F ST values is shown. (DOCX) [file pone.0143746.s013.docx]

**S10 Table: EASYPOP 2.0.1 simulations demonstrating the ability of the data to detect changes in genetic differentiation for several schemes of migration rate, generation time, and effective population size for a sample size of a) 10 birds per deme; b) 25 birds per deme; c) 50 birds per deme; and d) 200 birds per deme (for an effective population size of 400 only).** The mean, range, and standard deviation (SD) for *F*_ST_ represent that of 10 runs, and the number of those runs with significant *F*_ST_ values is shown.

a)

| **Migration Rate** | **0** | **0.01** | **0.1** |
| --- | --- | --- | --- |
| **150 generations (Ne = 400)** |  |  |  |
| *F*_ST_ (mean) | 0.327 | 0.068 | 0.005 |
| *F*_ST_ (range) | 0.273 - 0.376 | 0.039 - 0.114 | 0 - 0.022 |
| *F*_ST_ (SD) | 0.033 | 0.023 | 0.007 |
| Number of runs (out of 10) with p < 0.05 | 10 | 10 | 0 |
| **40 generations (Ne = 400)** |  |  |  |
| *F*_ST_ (mean) | 0.093 | 0.049 | 0.006 |
| *F*_ST_ (range) | 0.039 - 0.129 | 0.022 - 0.082 | 0 - 0.021 |
| *F*_ST_ (SD) | 0.027 | 0.020 | 0.008 |
| Number of runs (out of 10) with p < 0.05 | 10 | 9 | 0 |
| **40 generations (Ne = 100)** |  |  |  |
| *F*_ST_ (mean) | 0.309 | 0.176 | 0.012 |
| *F*_ST_ (range) | 0.200 - .378 | 0.113 - 0.314 | 0 - 0.030 |
| *F*_ST_ (SD) | 0.059 | 0.062 | 0.010 |
| Number of runs (out of 10) with p < 0.05 | 10 | 10 | 3 |
| **10 generations (Ne = 100)** |  |  |  |
| *F*_ST_ (mean) | 0.086 | 0.079 | 0.017 |
| *F*_ST_ (range) | 0.057 - 0.127 | 0.019 - 0.123 | 0 - 0.041 |
| *F*_ST_ (SD) | 0.022 | 0.032 | 0.015 |
| Number of runs (out of 10) with p < 0.05 | 10 | 10 | 3 |

b)

| **Migration Rate** | **0** | **0.01** | **0.1** |
| --- | --- | --- | --- |
| **150 generations (Ne = 400)** |  |  |  |
| *F*_ST_ (mean) | 0.326 | 0.063 | 0.004 |
| *F*_ST_ (range) | 0.246 - 0.394 | 0.026 - 0.083 | 0 - 0.020 |
| *F*_ST_ (SD) | 0.045 | 0.017 | 0.007 |
| Number of runs (out of 10) with p < 0.05 | 10 | 10 | 3 |
| **40 generations (Ne = 400)** |  |  |  |
| *F*_ST_ (mean) | 0.092 | 0.048 | 0.005 |
| *F*_ST_ (range) | 0.067 - 0.124 | 0.025 - 0.071 | 0 - 0.011 |
| *F*_ST_ (SD) | 0.016 | 0.013 | 0.004 |
| Number of runs (out of 10) with p < 0.05 | 10 | 10 | 2 |
| **40 generations (Ne = 100)** |  |  |  |
| *F*_ST_ (mean) | 0.306 | 0.175 | 0.014 |
| *F*_ST_ (range) | 0.221 - 0.374 | 0.092 - 0.275 | 0.009 - 0.032 |
| *F*_ST_ (SD) | 0.050 | 0.053 | 0.008 |
| Number of runs (out of 10) with p < 0.05 | 10 | 10 | 8 |
| **10 generations (Ne = 100)** |  |  |  |
| *F*_ST_ (mean) | 0.100 | 0.081 | 0.017 |
| *F*_ST_ (range) | 0.072 - 0.129 | 0.054 - 0.097 | 0.001 - 0.034 |
| *F*_ST_ (SD) | 0.016 | 0.014 | 0.011 |
| Number of runs (out of 10) with p < 0.05 | 10 | 10 | 9 |

c)

| **Migration Rate** | **0** | **0.01** | **0.1** |
| --- | --- | --- | --- |
| **150 generations (Ne = 400)** |  |  |  |
| *F*_ST_ (mean) | 0.324 | 0.060 | 0.004 |
| *F*_ST_ (range) | 0.251 - 0.391 | 0.021 - 0.091 | 0 - 0.011 |
| *F*_ST_ (SD) | 0.044 | 0.021 | 0.004 |
| Number of runs (out of 10) with p < 0.05 | 10 | 10 | 4 |
| **40 generations (Ne = 400)** |  |  |  |
| *F*_ST_ (mean) | 0.094 | 0.048 | 0.005 |
| *F*_ST_ (range) | 0.066 - 0.125 | 0.028 - 0.068 | 0 - 0.011 |
| *F*_ST_ (SD) | 0.016 | 0.012 | 0.003 |
| Number of runs (out of 10) with p < 0.05 | 10 | 10 | 7 |
| **40 generations (Ne = 100)** |  |  |  |
| *F*_ST_ (mean) | 0.301 | 0.182 | 0.016 |
| *F*_ST_ (range) | 0.231 - 0.364 | 0.112 - 0.264 | 0.008 - 0.033 |
| *F*_ST_ (SD) | 0.046 | 0.050 | 0.007 |
| Number of runs (out of 10) with p < 0.05 | 10 | 10 | 10 |
| **10 generations (Ne = 100)** |  |  |  |
| *F*_ST_ (mean) | 0.102 | 0.074 | 0.017 |
| *F*_ST_ (range) | 0.083 - 0.131 | 0.050 - 0.086 | 0.005 - 0.028 |
| *F*_ST_ (SD) | 0.013 | 0.013 | 0.009 |
| Number of runs (out of 10) with p < 0.05 | 10 | 10 | 10 |

d)

| **Migration Rate** | **0** | **0.01** | **0.1** |
| --- | --- | --- | --- |
| **150 generations (Ne = 400)** |  |  |  |
| *F*_ST_ (mean) | 0.325 | 0.057 | 0.005 |
| *F*_ST_ (range) | 0.267 - 0.377 | 0.027 - 0.080 | 0.003 - 0.008 |
| *F*_ST_ (SD) | 0.036 | 0.017 | 0.002 |
| Number of runs (out of 10) with p < 0.05 | 10 | 10 | 10 |
| **40 generations (Ne = 400)** |  |  |  |
| *F*_ST_ (mean) | 0.093 | 0.049 | 0.004 |
| *F*_ST_ (range) | 0.063 - 0.117 | 0.031 - 0.067 | 0.002 - 0.007 |
| *F*_ST_ (SD) | 0.016 | 0.013 | 0.001 |
| Number of runs (out of 10) with p < 0.05 | 10 | 10 | 10 |
